# Examination of the Combined Effects of Chondroitinase ABC, Growth Factors and Locomotor Training following Compressive Spinal Cord Injury on Neuroanatomical Plasticity and Kinematics

**DOI:** 10.1371/journal.pone.0111072

**Published:** 2014-10-28

**Authors:** Olivier Alluin, Hugo Delivet-Mongrain, Marie-Krystel Gauthier, Michael G. Fehlings, Serge Rossignol, Soheila Karimi-Abdolrezaee

**Affiliations:** 1 Multidisciplinary Team in Locomotor Rehabilitation of the Canadian Institutes of Health Research (CIHR) and Groupe de Recherche sur le Système Nerveux Central (GRSNC) of the Fonds de Recherche du Québec – Santé (FRQS), Department of Physiology, University of Montreal, Montreal, Quebec, Canada; 2 The Department of Physiology and Pathophysiology and the Spinal Cord Research Center, University of Manitoba, Winnipeg, Manitoba, Canada; 3 Department of Surgery and Spinal Program, University of Toronto; Toronto Western Research Institute, University Health Network, Toronto, Ontario, Canada; 4 Regenerative Medicine Program and Manitoba Institute of Child Health, Winnipeg, Manitoba, Canada; Rutgers-Robert wood Johnson Medical School, United States of America

## Abstract

While several cellular and pharmacological treatments have been evaluated following spinal cord injury (SCI) in animal models, it is increasingly recognized that approaches to address the glial scar, including the use of chondroitinase ABC (ChABC), can facilitate neuroanatomical plasticity. Moreover, increasing evidence suggests that combinatorial strategies are key to unlocking the plasticity that is enabled by ChABC. Given this, we evaluated the anatomical and functional consequences of ChABC in a combinatorial approach that also included growth factor (EGF, FGF2 and PDGF-AA) treatments and daily treadmill training on the recovery of hindlimb locomotion in rats with mid thoracic clip compression SCI. Using quantitative neuroanatomical and kinematic assessments, we demonstrate that the combined therapy significantly enhanced the neuroanatomical plasticity of major descending spinal tracts such as corticospinal and serotonergic-spinal pathways. Additionally, the pharmacological treatment attenuated chronic astrogliosis and inflammation at and adjacent to the lesion with the modest synergistic effects of treadmill training. We also observed a trend for earlier recovery of locomotion accompanied by an improvement of the overall angular excursions in rats treated with ChABC and growth factors in the first 4 weeks after SCI. At the end of the 7-week recovery period, rats from all groups exhibited an impressive spontaneous recovery of the kinematic parameters during locomotion on treadmill. However, although the combinatorial treatment led to clear chronic neuroanatomical plasticity, these structural changes did not translate to an additional long-term improvement of locomotor parameters studied including hindlimb-forelimb coupling. These findings demonstrate the beneficial effects of combined ChABC, growth factors and locomotor training on the plasticity of the injured spinal cord and the potential to induce earlier neurobehavioral recovery. However, additional approaches such as stem cell therapies or a more adapted treadmill training protocol may be required to optimize this repair strategy in order to induce sustained functional locomotor improvement.

## Introduction

Spinal cord injury (SCI) results in motor deficits below the level of injury that can be temporary or permanent, incomplete or complete depending on the severity of the lesion [1]–[4]. Recovery of locomotion is generally limited in SCI patients in spite of recent advances in clinical care and rehabilitation medicine [5]. Over the past years, various cellular and neurochemical repair strategies have been evaluated in experimental models of SCI for their efficacy in promoting neuroplasticity, axon regeneration, remyelination, and re-establishment of spinal circuitry to improve motor recovery following such injury [6]–[13]. Among these treatment strategies, targeting the inhibitory properties of chondroitin sulfate proteoglycans (CSPGs) located in the extracellular matrix of glial scar has shown promising potential in enhancing SCI repair [11], [12], [14]–[21].

Chondroitinase ABC (ChABC) facilitates the degradation of CSPGs in the injured spinal cord [12]. Therefore, over the past decade, ChABC has been utilized extensively in different models of SCI and in various combinatorial approaches to evaluate its impact in promoting functional repair and recovery [11], [14], [16], [20], [22], [23]. Emerging evidence demonstrates that ChABC alone or in conjunction with growth factors, neurotrophins and/or cell-based treatments can promote structural repair and regeneration in the injured spinal cord [11]–[15], [17], [21], [22], [24], [25]. ChABC treatment has also shown the potential to enhance locomotion in combination with other therapies in models of transection or compressive/contusive SCI [11], [14], [21].

ChABC in combination with cell therapies improves moderate recovery of function [11], [14]. Studies by Fouad and colleagues showed that ChABC in synergy with a Schwann cell bridge and transplantation of olfactory ensheathing cells allowed recovery of function in rats after complete transection SCI [14]. We have also shown that degradation of CSPGs in the glial scar with ChABC was needed to improve the outcomes of transplanting neural precursor cells (NPCs) in chronic SCI [11] or efficient activation and oligodendrocyte replacement of endogenous spinal cord precursor cells in subacute SCI [15]. Sustained delivery of ChABC in combination with a growth factor (GF) cocktail containing EGF, FGF2 and PDGF-AA significantly increased the long-term survival and migration of transplanted NPCs in chronic compressive SCI [11]. Combined effects of ChABC, GFs and NPCs transplantation attenuated axonal die back in the corticospinal tract (CST) and enhanced sprouting of the CST and serotonergic fibers in the chronically injured spinal cord [11]. Moreover, we have shown that ChABC and GFs synergistically enhances the activation and oligodendrocyte differentiation of endogenous precursor cells after SCI and attenuated astrogliosis [15]. However, our functional testing showed that although ChABC, by itself, allowed substantial structural plasticity in the spinal cord, it was not sufficient to enhance locomotor recovery until it was combined with NPCs transplantation and *in vivo* infusion of GFs [11].

ChABC has been also used in combination with motor training in order to synergistically enhance activity dependent neuroplasticity and the recovery of locomotion in experimental models of SCI [16], [20], [26]. Current evidence indicates a limited efficiency of motor training in improving function in rodents or cats with incomplete SCI; contusive/compressive or hemisection [26]–[29]. In our recent work, kinematic analyses showed that regular motor training on treadmill did not improve the quality of spontaneous recovery of locomotion in rats with incomplete compressive SCI, although the rats regained impressive functional locomotion of the hindlimbs [27].

The aim of the present study was to evaluate whether combination of ChABC and growth factors (GFs) with daily treadmill training would synergistically enhance endogenous repair mechanisms and activity dependent functional plasticity, together allowing a significantly better recovery of hindlimb locomotion in rats with compressive SCI. We used neuroanatomical and kinematic analyses to assess neuroplasticity and functional recovery over a 7-week recovery period. Our findings show that this strategy promoted a significant degree of structural plasticity in corticospinal and serotonin-dependent pathways and that our pharmacological treatment mitigated the evolution of chronic peri-lesional astrogliosis and inflammation with a modest synergistic effects of treadmill training. Although an earlier return of locomotion was associated with an overall improvement of hindlimb angular excursions in the group treated with ChABC + GFs, this observation did not reach the statistically significant threshold needed to claim a difference between groups. Additionally, we observed an impressive locomotor recovery at the endpoint of study in all rats; however, the combinatorial approach had no additive long-term beneficial effects on the locomotor parameters studied with our stringent kinematic parameters.

## Materials and Methods

All the neurobehavioural and neuroanatomical protocols in this study were performed based on approaches previously described by our group [6], [11], [15], [27], . We used appropriate randomization and blinding in all neurobehavioural and histological techniques. Unbiased methodologies were used to undertake the neuroanatomical assessments. A list of abbreviations is provided in Table 1.

**Table 1:** List of abbreviations.

ChABC	Chondroitinase ABC
GFs	Growth factors (EGF + FGF-2+ PDGF-AA)
SCI	Spinal cord injury
CSPGs	Chondroitin Sulfate Proteoglycans
BBB	Basso, Beattie and Bresnahan rating scale
LFB/HE	Luxol Fast Blue and hematoxylin/Eosin
BDA	Biotin dextran amine
CST	Corticospinal tract
CV	Coefficient of Variation
PBS	Phosphate Buffer Saline
PFA	Paraformaldehyde
GFAP	Glial Fibrillary Acidic Protein
5-HT	Serotonin
ANOVA	Analyse Of Variance
SEM	Standard Error of the Mean
F subphase	First part of the swing phase (Flexion)
E1 subphase	Second part of the swing phase (Extension)

### Animal care

A total number of 48 adult female Wistar rats (250–275 g) from Charles River Laboratory (Quebec, Canada) were used for different aspects of this study. Animals were housed in standard plastic cages at 22°C before spinal cord injury (SCI) and 25°C after SCI in a 12∶12 h light/dark photoperiod. Food (Agribrands Purina, Ontario, Canada) and drinking water were available *ad libitum*. Hardwood sawdust bedding (PWI brand, Quebec, Canada) was used before SCI and then was replaced by soft paper bedding (Diamond Soft Bedding, Harlan Teklad) after SCI to prevent skin lesion. Animals were examined daily. After SCI, the bladder was expressed two times daily until the recovery of spontaneous bladder function occurred (between 7 and 14 days post-injury). All the animal procedures included in the present study were approved by the University of Manitoba and the University of Montreal Research Ethics Board and were conducted according to the Canadian Guide to the Care and Use of Experimental Animals (Canadian Council on Animal Care).

### Experimental design and groups

After a one-week period of housing in the animal facility, rats were habituated to walk consistently on the treadmill at different speeds for three weeks. Afterwards, kinematic baselines were recorded for all rats during a second three-week period, and then SCI procedure was performed on each rat. One rat died during the surgery because of respiratory failure. Four days after SCI, rats were randomized into four experimental groups: 1) vehicle/untrained group (n = 12), 2) vehicle/trained group (n = 12), 3) ChABC+GFs/untrained group (n = 11), and 4) ChABC+GFs/trained group (n = 12). All SCI rats from groups 3–4 and 10 SCI rats from groups 1–2 (5 per group) underwent a second surgical procedure to infuse ChABC+GFs or vehicle via an intrathecal catheter connected to an Alzet osmotic minipump. After recovering from the implantation (2–3 days), kinematics was recorded weekly during 7 weeks for all rats of groups 3 and 4 and for 10 rats in groups 1 and 2 (5 per group). Finally, all the spinal cords were harvested for histology. Except for treadmill training sessions and data recording, all rats could move freely in their cage.

### Treadmill apparatus and habituation

The treadmill belt was laid under a Plexiglas box (Length = 41 cm×Depth = 9.3 cm×Height = 14 cm) with a removable top. All rats were progressively familiarized to walk consistently on the treadmill at speeds ranging from 14 m.min^–1^ (low) to 30 m.min^–1^ (high) during the three weeks training program before SCI. Each session lasted 15 minutes and the number of sessions depended on the ability of each rat to perform the task. A soft plastic stick was used when necessary to stimulate the rats and induce appropriate locomotion by touching their hindquarters. Progressively, rats learned to walk freely and regularly in the locomotor device, using all four limbs at each speed of the range defined above.

### Surgical procedures

All surgeries were performed in sterile conditions and under general gas anesthesia consisting in a mixture of O_2_/isoflurane (2%) given through a mask integrated in a surgical stereotaxic frame. Immediately after surgery, rats were placed under a heating lamp until they fully recovered consciousness. Animals were given postoperative analgesia (50 µg/kg Temgesic, Schering-Plough, Hertfordshire, UK) and saline (3 ml) subcutaneously to prevent pain and dehydration, and then received antibiotics in drinking water (Clavamox drops, Pfizer Animal Health) for 1 week.

#### Thoracic Spinal Cord Injury

The aneurysm clip spinal compression model has been extensively described previously by our group [6], [11], [15], [27], [30]–[32]. Briefly, a midline incision was made at the thoracic vertebrae (T5-T9) and the skin and superficial muscles were retracted. The rats underwent a T6–T8 laminectomy and then, received a 23.8 g clip (Walsh Inc., Oakville, Ontario, Canada) compression injury for 1 min at the level of T7 of the spinal cord. Next, a piece of sterile absorbable gelatin sponge (1.5×1×0.7 cm, Gelfoam, Pfizer Inc.) was placed over the dura between T6–T8 and finally, muscles and skin were sutured. This injury produces moderately severe incomplete SCI with neurological outcomes of spastic paraparesis.

#### Administration of ChABC and Growth Factors

At four days post SCI, all rats were anesthetized using a mixture of O_2_/isoflurane (2%) and then the injured spinal cord was carefully re-exposed using microsurgical techniques. ChABC (Seikagaku Corporation, Tokyo, Japan, 5 U/ml in saline plus 0.1% rat serum albumin) plus a cocktail of growth factors [GFs, including PDGF-AA (Sigma, 1 µg/100 µl), bFGF (Sigma, 3 µg/100 µl) and EGF (Sigma, 3 µg/100 µl) in a solution containing saline and 0.1% rat serum albumin] was infused for seven days using a subarachnoid catheter (Alzet, Rat IT, 0007741, 0.36 mm OD; 0.18 mm ID) connected to an osmotic minipump (Alzet pump model No.1007D, 0.5 µl/hr), as we have previously reported [6], [33]. The catheter was inserted in the subarachnoid space around the injured area. Five rats in each vehicle group underwent identical surgical procedures but received saline plus 0.1% rat serum albumin). In our previous studies in the same model of SCI [11], [15], we have extensively tested the efficacy of ChABC treatment in CSPG degradation. Using quantitative immunohistochemistry and slot blotting for CSPGs as well as 2B6 and C4S (detecting the degraded products of CSPGs), we have confirmed that one week ChABC treatment delivered at 3 days or 6 weeks post SCI can significantly degrade CSPGs after SCI. In our previous studies, our analysis at the end of one-week ChABC delivery with Alzet pump infusion has consistently confirmed a 60–75% reduction in CSPGs deposits and instead an 80% increase in C4S immunoreactivity (by-products of ChABC activity) in the matrix of subacute or chronic SCI [11], [15].

#### BDA Anterograde tracing of the corticospinal tract (CST)

Two weeks before the end of experiments, rats underwent anterograde tracing of the corticospinal tract (CST) with biotinylated dextran amine (BDA) (n = 3–6 rats/group). Under isoflurane anesthesia (as above), rats were positioned in a stereotaxic frame. BDA (10%, 10,000 MW; Invitrogen, Eugene, OR) was injected unilaterally into the left sensorimotor cortex at eight sites (0.5 µl per site) using the following coordinates (in reference to Bregma): (1) 1 mm anterior and 1 mm lateral; (2) 0.5 mm anterior and 1 mm lateral; (3) 1 mm posterior and 1 mm lateral; (4) 2.5 mm posterior and 1 mm lateral; (5) 0.5 mm posterior and 2 mm lateral; (6) 1.5 mm posterior and 2 mm lateral, (7) 1 mm anterior and 2.5 mm lateral, (8) 1.5 mm anterior and 2.5 mm lateral. Injections were made 1.2 mm from the surface of the cortex. Our previous studies have shown that the two weeks interval between BDA labeling and animal sacrifice is sufficient for anterograde labelling of the CST in thoracic regions [11], [34].

### Treadmill training program and kinematic recordings

Rats from groups 2 and 4 were all trained to walk on the motorized treadmill belt from 1 day to 7 weeks following SCI. The training program was performed 5 days/week and consisted in a walking session of 10 min daily on the treadmill at various speeds (from 14 to 26 m.min^–1^) depending on the locomotor recovery level of each rat. Early after SCI, rats were incapable of autonomous hindlimb stepping, and were therefore stimulated by pinching the perineum. This evoked, in most cases, some hindlimb locomotion adapted to the belt speed but sometimes only flexion/extension alternation without plantar paw placement. During the training session, the belt speed was set at 14 m.min^–1^ and the trunk of the animal was manually maintained to limit the lateral imbalance as long as the animal required perineal stimulation to walk. As soon as the rat was capable to walk without perineal stimulation, the belt speed was incremented in steps of 2 m.min^–1^ every 2 minutes until the maximum speed tolerated by the rat was reached (*i.e.* in the range defined above). Throughout the recovery period, the trunk of the animals was manually supported when necessary.

#### Kinematic recordings

Kinematic baseline data were recorded at 14, 20 and 26 m.min^–1^ for each rat to obtain control values. After SCI, the locomotor performance was evaluated weekly for 7 weeks until the maximum velocity that the animal could reach in the range defined above. Moreover, the untrained groups were assessed during short sessions to avoid the training effect of the evaluation itself. Kinematic recording method has been previously described in detail by our group [27]. Briefly, after shaving the left hindquarter of the rat, five markers were set on the skin of the lateral side at the level of ilium, greater trochanter, ankle joint, metatarsophalangeal joint and tip of the third toe. During the recording session, a left side view of the rat walking on the treadmill was captured using a high frequency numerical video camera (120 Hz). All the kinematics parameters were generated from the *x, y* coordinates of each marker and from the paw contact/lift events. Triangulation was used to extract the knee position from hip and ankle joint markers.

#### Definition of locomotion on treadmill

Since rats in each group exhibited different locomotor capability on treadmill at the end of the experiment, it was necessary to define simple criteria to include in kinematics analysis only animals capable of producing motor pattern considered as locomotor. Rats capable of performing at least 10 consecutive bilateral flexion-extension alternations of the hindlimbs with paw placement (plantar or dorsal) on the treadmill belt at 14 m.min^–1^ were included in locomotion analysis. Rats that have not reached these criteria were considered not to have recovered locomotion.

#### Calculation of coordination

The method employed to evaluate the interlimb coordination has been described in detail previously [27] and gives an index of the neuronal coupling between each paw during locomotion. The coordination between fore- and hindlimbs (*i.e.* anteroposterior) as well as limbs from the same girdle (*i.e.* homologous) was calculated using the same method. Briefly, the coordination value of a given limb in a given step cycle represents the temporal position of the ground contact from this limb relative to the whole step cycle duration of the other limb. For instance, left hindlimbs homologous coordination value of 0.5 in a given step cycle means that ground contact of left hindlimb occurs at 50% of the right hindlimb step cycle duration.

#### Evaluation of individual variability

The method of individual variability assessment has been previously reported in detailed description [27]. Briefly, the coefficient of variation (CV) of every kinematic parameter was used to assess the intrinsic variability of each rat during locomotion. Since the analysis of locomotion of a given rat was based on consecutive sequences of several step cycles, each individual kinematic data at a given time point was an averaged value from the consecutive step cycles associated with a standard deviation. Thus, in the present study, the CV of a given parameter in a given rat at a given time point is the individual standard deviation expressed in percent of the mean of this parameter.

### Tissue Processing and Neuroanatomical Analyses

#### Animal perfusion

At the end of experiments, animals were deeply anesthetized with sodium pentobarbital (80 mg/kg, intraperitoneal.) and then perfused transcardially with cold phosphate buffer saline (PBS, 0.1 M) followed by 4% paraformaldehyde (PFA) in 0.1 M PBS, pH 7.4. A two cm length of the spinal cord centered at the injury center was dissected and processed for different procedures as follows:

#### Frozen Sections

For cryotomy, the spinal cord were post-fixed in the perfusion solution plus 10% sucrose overnight at 4°C, and then cryoprotected in 20% sucrose in PBS for 48 hr at 4°C. Then, the spinal cord centered at the injury site was dissected and embedded in mounting media (HistoPrep, Fisher Scientific) on dry ice. Cryostat sections (25 µm) were cut and stored at –80°C.

#### Vibratome sectioning

The spinal cords of the rats that underwent BDA anterograde labeling were postfixed in 4% PFA for overnight at 4°C, and then stored in PBS containing 0.1% sodium azide. The spinal cord were embedded in 10% low-temperature gelling agarose (Sigma), and 50 µm free floating serial transverse sections were cut on a vibratome (Leica) and collected in multi-well plates containing PBS plus 0.1% sodium azide.

#### Morphometric assessment of spinal cord lesion

Serial frozen spinal cord sections at 500 µm intervals were stained with myelin-selective pigment Luxol Fast Blue (LFB) and the tissue stain Hematoxylin-Eosin (HE) to identify the injury epicentre (N = 3–6 rats/group) as we reported previously [27], [30]. Tissue sections displaying the largest proportion of cystic cavity compared with total cross-sectional area were taken to represent the epicentre of the injury. For analysis of tissue sparing and cavity formation after SCI, for each rat, we selected the spinal sections at epicentre and 1 mm and 2 mm rostral and caudal to the epicentre. The sections were stained with LFB-HE. We also immunostained the adjacent slide for astrocytes using an antibody against GFAP (rabbit, 1∶1000, Dako) to precisely identify the borders of lesion cavity in each section. Reactive astrocytes surround the lesion area that includes the tissue debris and macrophages/microglia. Therefore, we used this criterion to differentiate the damaged tissue/debris within the lesion from the surrounding spared spinal cord tissue. The measurements were carried out in a blinded manner on coded slides using NIH ImageJ software (Media Cybernetics Inc., MD). Spared tissue was measured and normalized as a percentage of the total cross-sectional area of the spinal cord.

### Immunohistochemical procedures and image analysis

For all immunohistochemical staining, the blocking solution contained 5% non-fat milk, 1% BSA and 0.3% Triton X-100 in 0.1 M PBS unless otherwise has been mentioned. The specificities of all antibodies were verified with both a negative control, omitting the primary antibody in our immunohistochemistry staining protocol, and a positive control, testing the antibody on tissues or cell preparations known to express the antigen.

#### GFAP and CD11b (OX42) immunostaining

Five transverse sections at epicentre and 1 mm and 2 mm rostral and caudal to the epicentre from each rat (N = 3–6 rats/group) were immunostained against GFAP and OX42 to assess astrocytic glial scar and macrophages/microglia, respectively. The frozen slides were air-dried at room temperature, and then were washed with PBS for 10 min. The sections were blocked and then incubated with primary antibodies. The following primary antibodies were used overnight at 4°C: rabbit anti-GFAP (1∶1000, Dako) for astrocytes and mouse anti-CD11b (OX42, Serotec, 1∶50). The slides were washed in PBS three times and then incubated with fluorescent Alexa 568 or 488 goat-anti mouse or anti-rabbit secondary antibodies (Invitrogen, 1∶500) for 1 hr. The slides were coverslipped with Mowiol mounting medium containing DAPI to counterstain the nuclei. The images were taken using a Zeiss 710 laser confocal microscope or a Zeiss AxioVision microscope.

#### BDA visualization

We employed anterograde BDA labeling to study the sprouting of the CST fibers in the spinal gray matter as we described previously [11]. Three transverse vibratome sections (50 µm thickness) per animal at 5, 9 and 11 mm rostral to the injury site were selected for immunohistochemical processing to visualize BDA (N = 3–5/rats per group). One section per distance was processed for immunohistochemistry. Free-floating sections were incubated with the following reagents: 0.3% hydrogen peroxide (H_2_O_2_) in absolute methanol for 30 min at 4°C (using blocking solution as above), Vectastain AB (ABC Elite Kit, Vector Labs) in PBS containing 0.5% Triton X-10 according to manufacturer instructions for 2 hrs, 0.05% diaminobenzidine and 0.05% H_2_O_2_ in PBS for about 5 min. Sections were mounted onto slides and air-dried. Then, the slides were dehydrated through an alcohol series, cleared in xylene and coverslipped with Permount (Fisher Scientific).

#### 5-HT immunostaining

Free-floating (50 µm thickness) at the injury epicenter and various distances relative to the injury epicenter: 1.5, 3 and 6 mm both rostrally and caudally were processed for immunohistochemistry for 5-HT (N = 3–6 rats/group). One section per distance was processed for immunohistochemistry. Sections were blocked and then incubated in anti-rabbit 5-HT antibody (1∶10,000, ImmunoStar) for overnight at 4°C, and then Alexa 568 goat-anti rabbit secondary antibody (1∶400, 2 hrs at room temperature) with three PBS washes after each step.

### Image processing and analysis

In all neuroanatomical procedures, quantification was executed in an unbiased fashion by examiners blinded to the treatment groups based on the previously described methods by our group [6], [11], [15], [27], [30].

#### Immunohistochemical assessments of GFAP, CD11b (Ox42) and 5-HT

For immunodensity measurements of GFAP, CD11b (Ox42) and 5-HT, we imaged the entire cross section of the spinal cord at 10× primary objective using Mosaic tiling software (Zeiss). Then, using NIH Image analysis system (Image J), we traced entire cross section of the spinal cord and measured the relative density of GFAP, CD11b or 5-HT immunoreactivity as we performed previously [11] (N = 3–6 rats/group). Furthermore, we performed automatic thresholding for each image using NIH ImageJ software to determine the threshold for specific signal. After setting the threshold, the immunodensity above the threshold was automatically calculated. Background intensity from an area with no positive immunoreactivity was also subtracted from the intensity value to correct for non-specific reactions. Then, we divided the integrated density to the sample area to calculate the mean density per unit area. This calculation was performed to compensate for the different size of the region of interest in the spinal cord as we performed previously [11].

#### Assessment of axonal sprouting in the corticospinal tract

We performed anterograde BDA labeling to assess the sprouting of the CST fibers in the spinal gray matter as we described previously [11]. We normalized the intensity value of the BDA labeled collaterals in the gray matter to the intensity of BDA labeled fibers in the main CST to correct for inter-animal variation in the BDA labeling efficiency and/or the preservation of CST fibers. We imaged the dorsal columns of the spinal cord at 10×primary magnification using a Zeiss microscope at 5, 9 and 11 mm rostral to injury epicenter (N = 3–5/rats per group). In our SCI model, descending fibers of the CST are directly impacted by the injury and undergo Wallerian degeneration at and caudal to the injury epicenter. The axons in the rostral segment are also subjected to retrograde axonal degeneration or “die-back” as we described before [11].

Therefore, we selected rostral distances at 5 mm and further to the injury epicenter for our assessment of the CST fibers collateral sprouting. Using ImageJ Software, we measured the relative density of BDA immunoreactivity in the CST within the dorsal column. We employed automatic thresholding for each image using ImageJ software to determine the threshold for specific signal. The immunodensity above the threshold was quantified and the mean net density for BDA was calculated by dividing the immunointensity for BDA to the traced area. Background intensity from an area with no BDA positive immunoreactivity was also subtracted from the intensity value to correct for non-specific reactions. For collateral sprouting in spinal gray matter, we undertook the same approach as described above for BDA in the main CST to measure the relative intensity of BDA labeling within a the dorsal and intermediate gray matter. Then, we normalized the intensity value of the BDA labeled collaterals in the gray matter to the intensity of BDA labeled fibers of the main CST [11].

### Statistical analysis

For neuroanatomical examinations and statistical intensity measurements, Two-Way ANOVA comparing groups and distances was used followed by post hoc pairwise multiple comparisons testing by the Holm-Sidak post hoc test. For kinematic analysis, individual data from each rat at every time point were averaged from a minimum of 10 consecutive locomotor cycles. Analysis of kinematic and regression data was performed using SigmaPlot program (Systat software Inc., San Jose, USA). Two-way ANOVA Repeated Measures was used to compare the effects of groups and delays on kinematics parameters followed by all pairwise multiple comparison (Holm-Sidak post hoc test) when ANOVA was significant. ANOVA was also used to test the linear regression between kinematic and neuroanatomical data. All the regressions analysis presented in this study have passed the normality test (Shapiro-Wilk) and the equal variance test. Non-parametric χ^2^ with Yates correction and power test (α set at 0.05) or Fisher’s exact test analysis were used to compare the distribution of rats capable or not to walk on treadmill at the different time points and velocity after SCI. Samples sizes were calculated to allow a 20% chance for a Type II error (β ≤0.2). Grouped data are reported as mean ± SEM and the significance threshold for all statistical analysis was *p*≤0.05.

## Results

### Morphometric analysis of the spinal cord lesion

**Figure 1:**
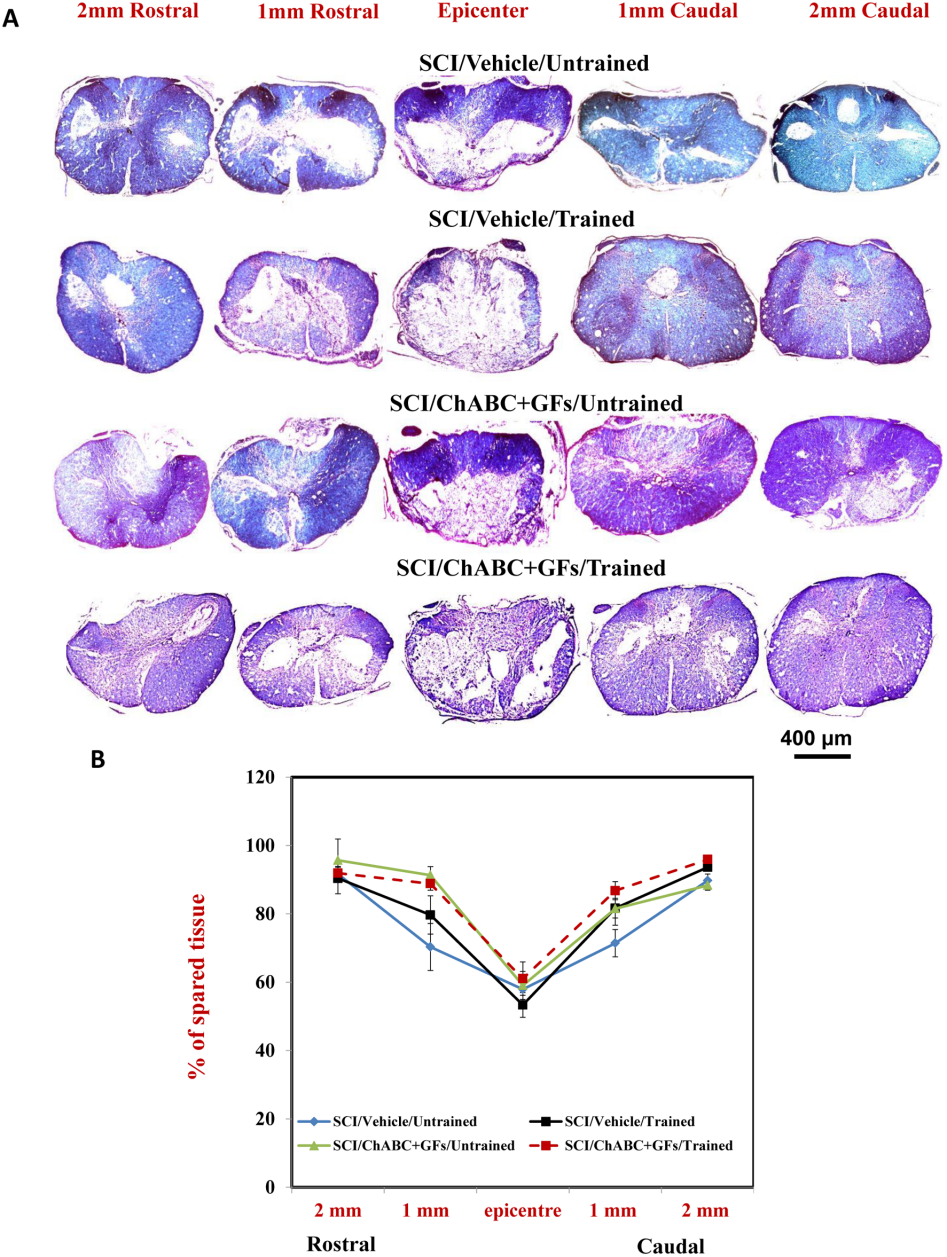
Morphometric analysis of the spinal cord lesion. (**A**) LFB/HE staining of cross sections of the injured spinal cord at various distances to the injury epicentre (both rostrally and caudally) is depicted for all experimental groups at seven weeks post-injury. The area of spared spinal cord tissue was traced and measured. (**B**) The percentage of spared tissue was calculated by normalizing the area of spared tissue to the total cross sectional area of the spinal cord. Although our quantitative analysis showed a positive trend in increasing tissue preservation in groups that received ChABC+GFs/Trained and ChABC+GFs/Untrained compared to Vehicle/Untrained counterpart in 1 mm rostral area, the difference was not statistically significant (Fig. 1B, Two-Way ANOVA, N = 3–6/group).

We first studied the effects of the treatments on tissue preservation and lesion following SCI. We quantified the extent of tissue sparing in spinal cord serial sections at five distances along the rostrocaudal length of the injured spinal cord centered at the injury epicenter as we described previously [27], [30]. Representative photomicrographs of Luxol Fast Blue, hematoxylin and eosin-Y (LFB/HE) counterstained cross-sectional spinal cord sections are demonstrated for all treatment groups in Fig. 1A. Values representing the mean percentage of spared tissue for each examined distance in all experimental groups are summarized in Table 2. Our quantitative analysis showed no statistically significant difference in tissue sparing across the experimental groups suggesting that ChABC, GFs and training had no beneficial effects on attenuating tissue degeneration following injury.

**Table 2:** Percent of spared tissue.

Groups	2 mm rostral	1 mm rostral	Epicenter	1 mm caudal	2 mm caudal
**SCI/Vehicle/Untrained**	91.6±1.99	70.3±6.9	57.9±4.2	71.4±4	89.7±1.93
**SCI/Vehicle/Trained**	90.4±4.5	79.7±5.5	53.35±3.6	81.65±2.8	93.7±1
**SCI/ChABC+GFs/Untrained**	95.7±6.2	91.3±2.47	59.1±4.1	81.8±4.87	88.36±1.46
**SCI/ChABC+GFs/Trained**	91.9±1.95	88.8±1.91	61.03±4.8	86.8±2.65	95.91±0.6

*Values are expressed as mean ± SEM*.

### Effects of ChABC, growth factors and daily exercise on the evolution of chronic astrogliosis and inflammation around the SCI lesion

**Figure 2:**
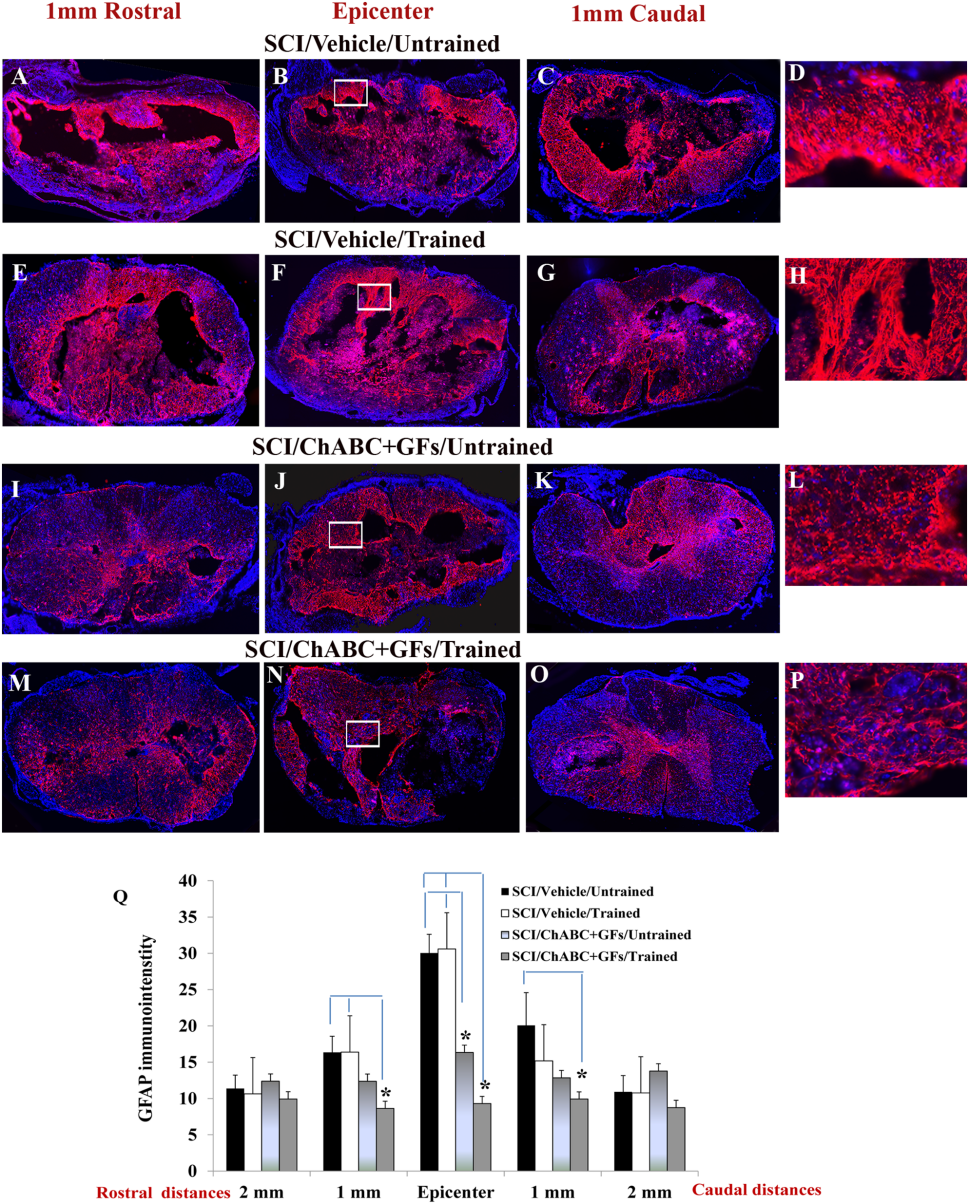
Effects of ChABC, GF and daily exercise on chronic astrogliosis after SCI. (**A–P**) Images show cross sections of the injured spinal cord immunostained for GFAP to mark astrocytes. Representative images from vehicle/untrained, vehicle/trained, ChABC+GFs/untrained and ChABC+GFs/trained injured rats are depicted at various rostral and caudal distances to the lesion epicenter. Confocal images show an overall reduction in the expression of GFAP particularly in the surrounding parenchymal region in ChABC+GFs/untrained and ChABC+GFs/trained groups relative to both Vehicle/Untrained and Vehicle/Trained groups. Images in D, H, L, and P depict magnified areas inside the boxed regions identified in B, F, J and N, respectively. (**Q**) Our quantitative analysis of GFAP immunointensity confirmed a significant reduction in astrogliosis in the ChABC+GFs/trained group at the SCI epicenter as well as 1 mm rostral and caudal in comparison to the Vehicle treated groups. ChABC+GFs/untrained group also demonstrated a significant decrease in GFAP immunoreactivity at the epicenter compared to both vehicle treated groups (Two-way ANOVA, *p<0.05, n = 3–6/group). Although ChABC+GFs/trained group consistently showed less astrogliosis compared to the ChABC+GFs/untrained counterpart, our statistical analysis showed no significant differences between the two groups.

We further investigated the influence of ChABC, GFs and daily treadmill training on the evolution of chronic reactive astrogliosis and inflammation within the spinal cord lesion. We quantified GFAP immunodensity (marking astrocytes) in spinal cord sections at the injury epicenter and at the peri-lesional areas around 1 mm and 2 mm rostral and caudal points to the epicenter (Fig. 2A–P, images depict representative sections at the epicenter and 1 mm rostral and caudal). Our data showed clear effects of ChABC and GFs attenuating astrogliosis, evident by reduced GFAP immunodensity, at the injury epicenter in the rats that received pharmacological treatments relative to untreated rats (Fig. 2Q, *p<0.05, two-way ANOVA, Holm-Sidak *post hoc*). Our analyses of the peri-lesional areas also showed a significantly reduced GFAP immunodensity in ChABC+GFs/trained group compared to the SCI vehicle/untrained group at 1 mm rostral and caudal points and compared to the SCI vehicle/trained group only at the 1 mm rostral distance (Fig. 2Q). These significant peri-lesional effects only present in ChABC+GFs/trained animals suggest a trend of synergy in our combinatorial strategy. Although there was a trend for reduced GFAP immunodensity in ChABC+GFs/trained group in comparison to the ChABC+GFs/untrained group, the difference between the two groups was not statistically significant. Interestingly, our examination also showed a significant attenuation in astrogliosis in ChABC+GFs/untrained group compared to the SCI vehicle/trained and SCI vehicle/untrained groups at the epicenter of the lesion (Fig. 2Q) which is in agreement with our previous results in a subacute SCI lesion after administration of ChABC+GFs [15]. Here, we did not observe any difference in lesional and peri-lesional astrogliosis between the two trained and untrained SCI vehicle groups as the result of treadmill motor exercise alone. Although, the differences between ChABC+GFs trained and untrained groups did not reach the significant level, the positive effects only obtained with the 3 combined treatments in the peri-lesional area suggest a potential moderate synergy. However, ChABC and GFs treatment seemed to be the key to induce the main effects.

**Figure 3:**
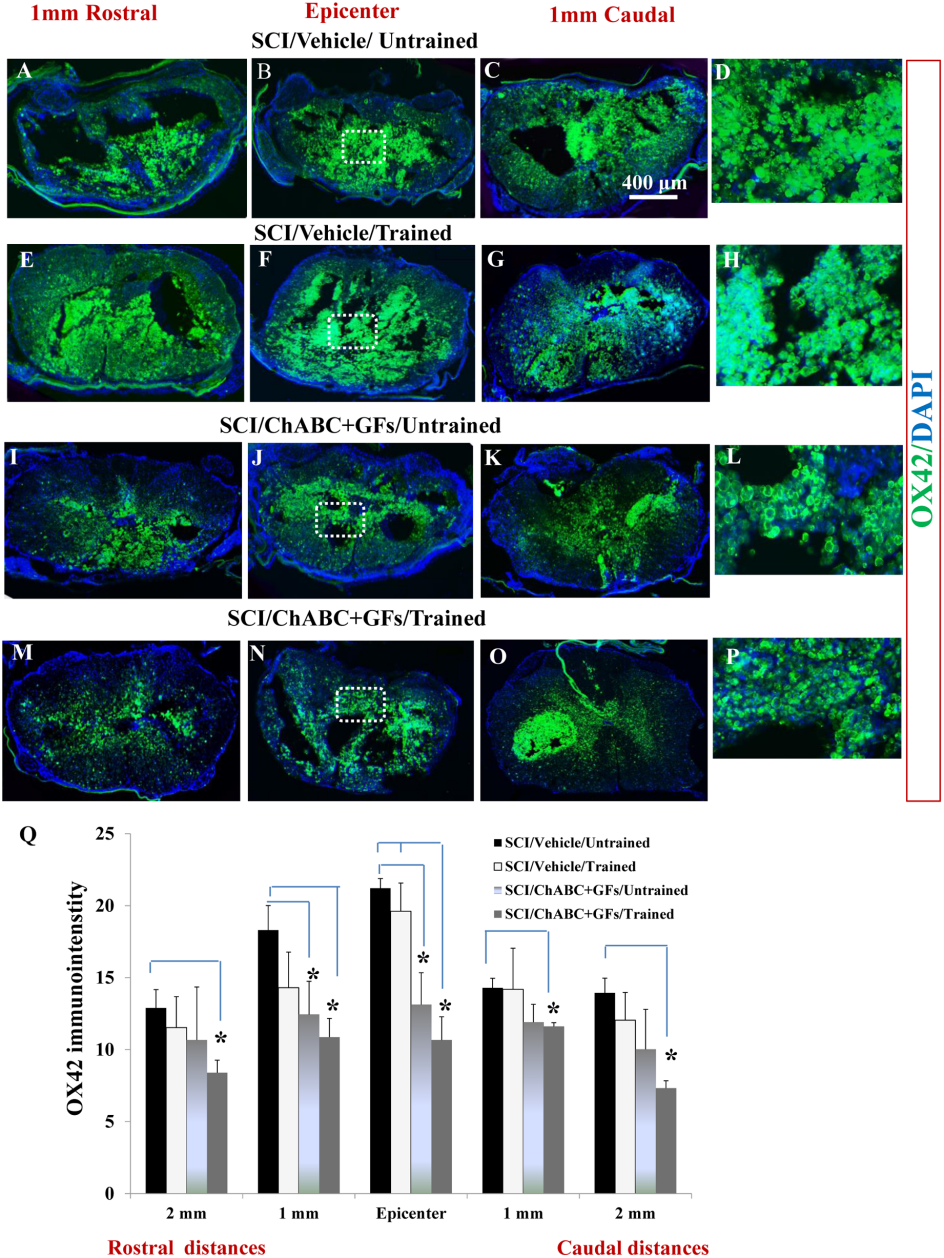
Effects of ChABC, GF and daily exercise on chronic presence of macrophages/microglia at the site of SCI lesion. (**A–P**) Confocal images showing the cross sections of the injured spinal cord immunostained for CD11b (OX42). Representative images from vehicle/untrained, vehicle/trained, ChABC+GFs/untrained and ChABC+GFs/trained injured rats are depicted at various rostral and caudal distances to the lesion epicenter. CD11b marks macrophages and microglia populations. Images in D, H, L, and P depict magnified areas inside the boxed regions identified in B, F, J and N, respectively. (**Q**) Our quantitative analysis of CD11b immunointensity showed reduced recruitment of macrophages/microglia in ChABC+GFs/trained group compared to the Vehicle/Untrained group at all distances and compared to the Vehicle/Trained group at the epicenter. Interestingly, ChABC+GFs/Untrained group also showed a significant reduction in CD11b immunoreactivity compared to the Vehicle/Untrained group at the injury epicenter (*p<0.05, Two way ANOVA, Holm-Sidak *post hoc,* N = 3–6/group).

Next, we studied how treatment with ChABC, GFs and training affect chronic inflammation assessed by the presence of macrophages/microglia within the lesional and peri-lesional regions (Fig. 3A–P, images depict representative sections at the epicenter and 1 mm rostral and caudal). Our neuroanatomical data at all the above-mentioned distances showed a significantly reduced macrophages/microglia in the chronic SCI lesion in the ChABC+GFs/trained group compared to the vehicle/untrained group and at the epicenter compared to the vehicle/trained group (Fig. 3R, *p<0.05, two-way ANOVA, Holm-Sidak *post hoc*). However, there was no significant difference between ChABC+GFs/trained and ChABC+GFs/untrained groups. Interestingly, rats in the ChABC+GFs/untrained group also showed less density of macrophages/microglia compared to the vehicle/untrained group that was significantly different at the epicenter and 1 mm rostral peri-lesional area, showing the effect of ChABC and GFs treatments on the reduction of inflammation process (Fig. 3Q, *p<0.05). This is also in agreement with our previous study that showed the positive effects of ChABC alone and in combination with GFs in attenuating lesional recruitment of macrophages/microglia in subacute SCI [15]. In the present chronic study, the significant reduction in macrophages/microglia at 2 mm rostral and 1 and 2 mm caudal to the epicenter only in ChABC+GFs/trained group suggests moderate synergistic effects of training with ChABC+GFs treatments.

### ChABC, growth factors and daily training synergistically promote collateral sprouting of the corticospinal tract and serotonergic fibers after SCI

**Figure 4:**
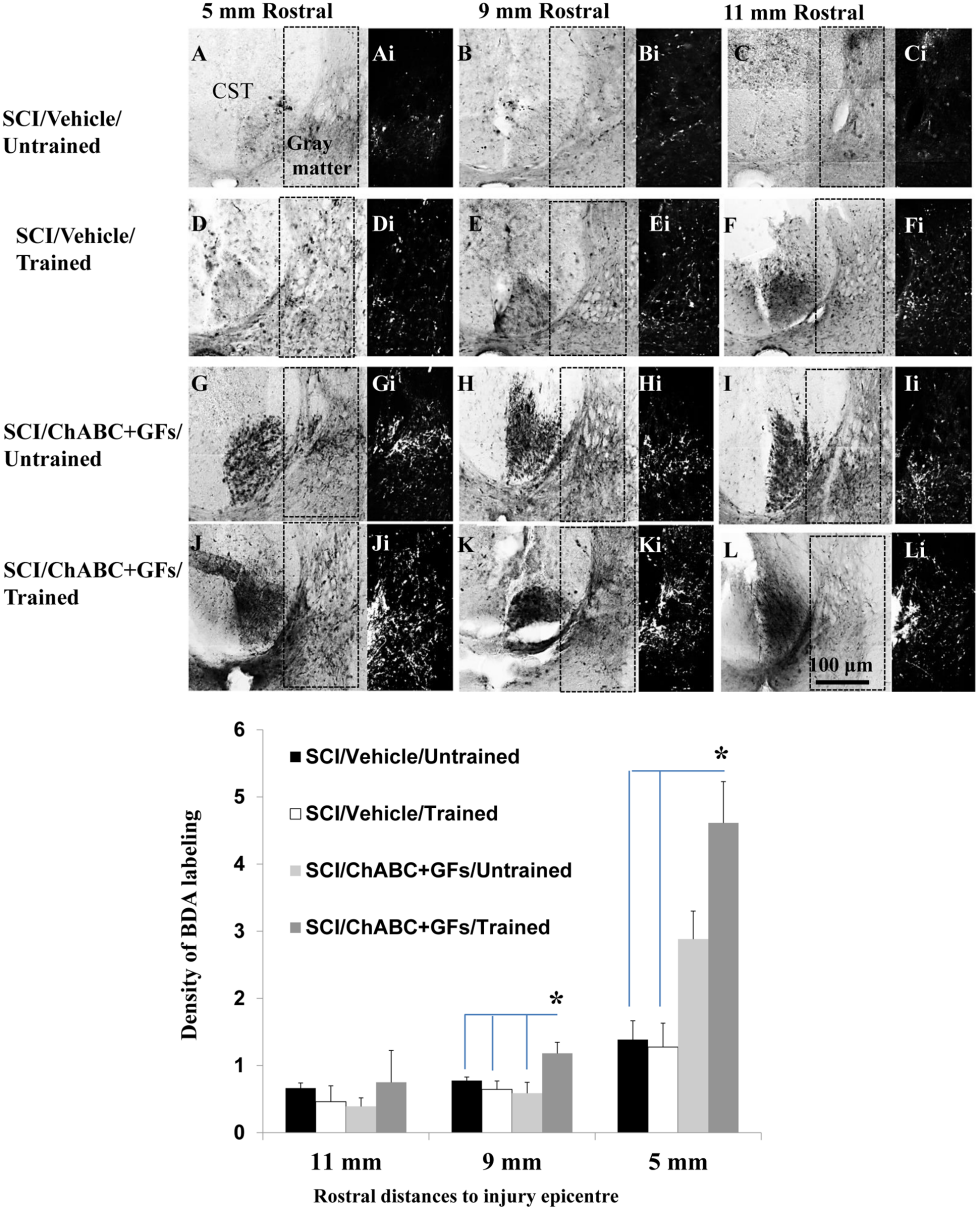
Synergistic effects of ChABC, GF and daily exercise enhance the collateral sprouting of the CST axons in the injured spinal cord. (**A–L**) Images of the BDA-labeled CST at 5, 9 and 11 mm rostral to the lesion are depicted in different experimental groups after unilateral injections of BDA into the sensorimotor cortex (Ai-Li). Inverted images were generated from the boxed region depicted in A–L for better visualization. BDA labeling was unilateral, so only contralateral CST is labeled. To correct for inter-animal variations in the BDA labeling efficiency, the intensity value of the BDA labeled collaterals in the gray matter were normalized to the intensity of BDA labeled fibers of the main CST. (**M**) Quantification of the areas depicted in Ai-Li at various distances revealed an increase in BDA density in the combined ChABC+GFs/trained treatment group compared to all other groups at 5 and 9 mm distances. Comparison of BDA-labeled collaterals among different injured groups also showed an increase in the density of BDA-labeled CST fibers in the ChABC+GFs/untrained group that was significantly higher than both vehicle treated groups at 5 mm rostral point to the SCI epicenter (*p<0.05, Two-way ANOVA, Holm-Sidak *post hoc*, N = 3–5).

To assess whether our combined strategy would enhance neuroplasticity in the injured spinal cord, we studied the sprouting of two major descending pathways to the spinal cord: the corticospinal tract (CST) and serotonergic pathway. For studying the CST, we used anterograde BDA tracing to label the CST and its collateral fibers in the spinal cord (Fig. 4A–L). Of note, as we have shown previously, in our protocol to induce clip compressive SCI, the CST is disrupted by the injury and its axons caudal to the injury epicenter undergoes degeneration [11]. Moreover, CST axons proximal to the impact of injury are subject to axonal die-back and therefore are absent in rostral distances closer to the epicenter [11], [35]. Accordingly, we examined the density of BDA labeling at starting at 5 mm distance rostral to the injury epicenter where the main CST is partially preserved following clip compression SCI [11]. To correct for inter-animal variation in the BDA labeling efficiency, we normalized the intensity of the BDA labeled collaterals in the gray matter to the intensity of BDA labeled main CST in the same section. Analysis of the dorsal and intermediate gray matter regions (boxed area depicted in Fig. 4A–L), at 5, 9 and 11 mm rostral points, revealed a significant increase in the density of BDA-labeled collaterals of the CST axons in the ChABC+GFs/trained group when compared to all other SCI experimental groups at 9 mm and to Vehicle/trained and untrained groups at 5 mm distances rostral to the lesion (Fig. 4M, *p<0.05, two-way ANOVA, Holm-Sidak *post hoc*). While the ChABC and GFs treatments together also demonstrated a non-significant trend toward a positive effect on promoting collateral sprouting of the CST in its target region at 5 mm rostral point, only the combination of these treatments with training was sufficient to significantly promote CST sprouting. Interestingly, in agreement with our previous reports with ChABC treatment, our combined strategy did not result in long distance axonal regeneration in the CST, as we did not detect any BDA traced CST fibers beyond the lesion [11].

**Figure 5:**
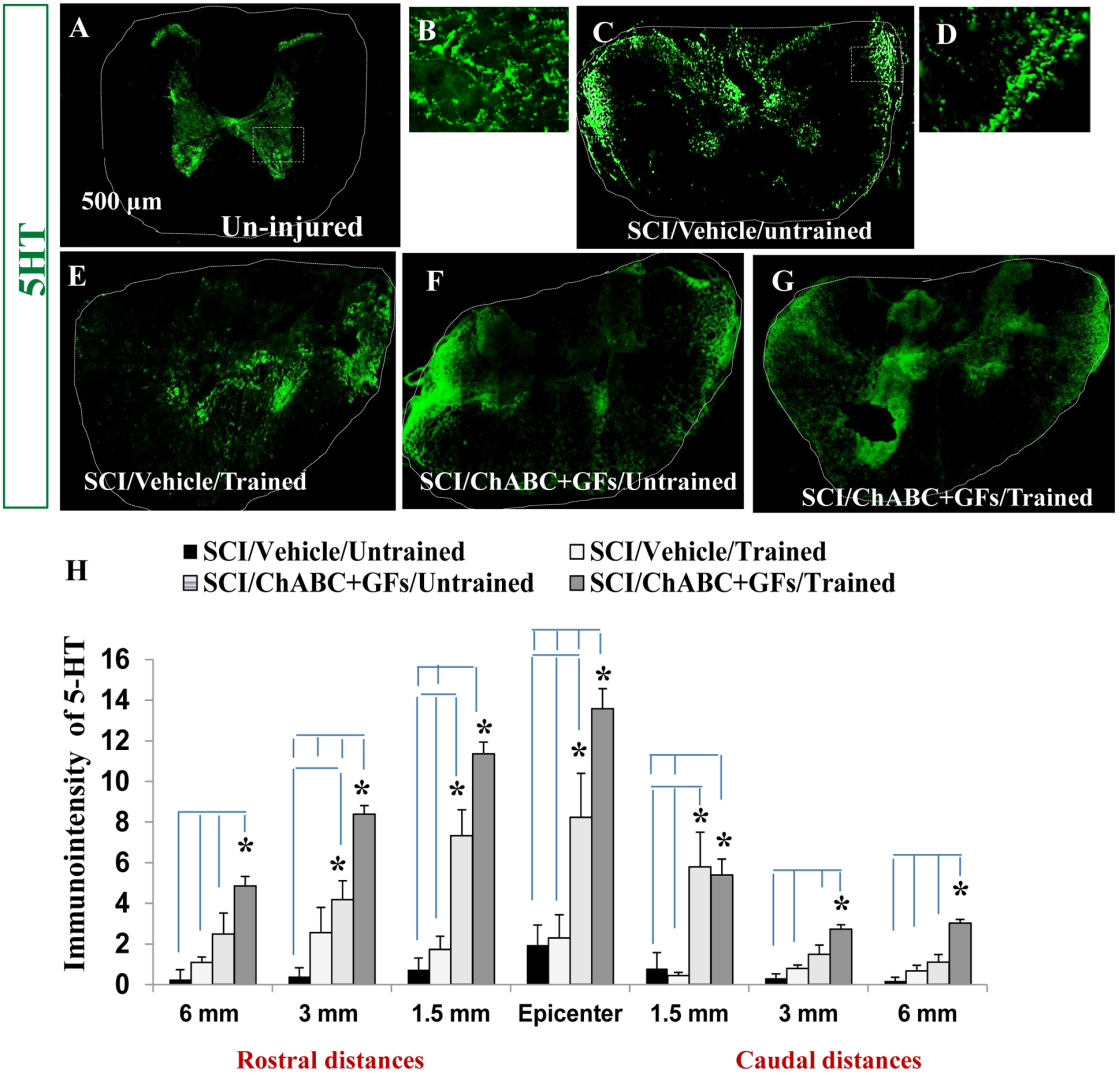
Combination of ChABC, GFs and training promotes plasticity of serotonergic fibers after SCI. (**A**) Transverse section of an uninjured spinal cord at mid-thoracic region demonstrates normal innervation pattern of serotonergic pathway (5-HT positive fibers) within the spinal cord. (**B**) Higher magnification of the boxed area in A shows the presence of serotonergic fibers in the gray matter areas representing of the signal that was quantified in our assessments. (C–G) At 7 weeks post-injury, 5-HT positive fibers in all experimental groups show significant changes in their localization (images shown for 1.5 mm rostral). In contrast to uninjured spinal cord, 5-HT immunoreactive fibers were sprouting in different regions of white matter in all injured groups. (**D**) Higher magnification of the boxed area in C shows the presence of serotonergic fibers in the white matter areas representing of the signal that was quantified in our assessments (H) Quantification of 5-HT immunointensity in the entire cross section of the spinal cord (traced areas in images) at various rostral and caudal distances revealed significantly increased level of 5-HT immunoreactivity in ChABC+GFs/trained group compared to both vehicle treated group at all examined rostral and caudal distances (*p<0.05, Two-way ANOVA, Holm-Sidak *post hoc*, n = 3–6). Interestingly, at the epicenter and 1.5 mm rostral and caudal distances, the ChABC+GFs/untrained group also showed a significantly higher expression of 5-HT-immunoreactivity compared to the vehicle treated groups (*p<0.05, Two-way ANOVA, Holm-Sidak *post hoc*).

We further examined the effects of ChABC+GFs/training on the plasticity of the descending serotonergic pathway that modulates neuronal activity and locomotion in the spinal cord [36]–[39]. In intact spinal cord, serotonergic fibers mainly terminate in the ventral and intermediate gray matter as well as lamina X and to lesser extent in the dorsal horn (Fig. 5A). As we reported previously, a sizable increase in serotonin immunoreactivity is seen in rostral white matter regions particularly in the lateral and dorsal funiculi in our base-line SCI model (Fig. 5C) suggestive of spontaneous plasticity, increased expression of serotonin and/or accumulation of 5-HT in the serotonergic axons. Assessment of 5-HT immunodensity in multiple rostral and caudal distances to the injury epicenter (Fig. 5H) showed a significant increase in the density of serotonergic axons in ChABC+GFs/trained group (Fig. 5G) at all examined distances compared to both vehicle/untrained and vehicle/trained groups (Fig. 5C and E, *p<0.05). In addition, the 5-HT immunodensity was significantly increased in ChABC+GFs treated group with treadmill training relative to the ChABC+GFs/untrained group (Fig. 5F, *p<0.05) at most distances excepted 1.5 mm rostral and caudal from the epicenter. On another hand, no difference was present between Vehicle/trained and Vehicle/untrained groups at all distances demonstrating a clear synergistic effect of the treadmill training with the pharmacological treatment toward an increase of 5-HT expression. Moreover, ChABC+GFs/untrained group showed a statistically significant elevation in 5-HT immunodensity at the epicenter as well as at 1.5 mm rostral and caudal points compared to both vehicle/untrained and vehicle/trained injured groups (Fig. 5H, *p<0.05). Serotonin density was also significantly higher at 3 mm rostral to the SCI epicenter in ChABC+GFs/untrained group compared to the vehicle/untrained counterpart. These findings collectively indicate that ChABC+GFs promote plasticity in serotonergic pathways and regular motor training enhances the magnitude of this response.

### Recovery of kinematic parameters during treadmill locomotion

**Figure 6:**
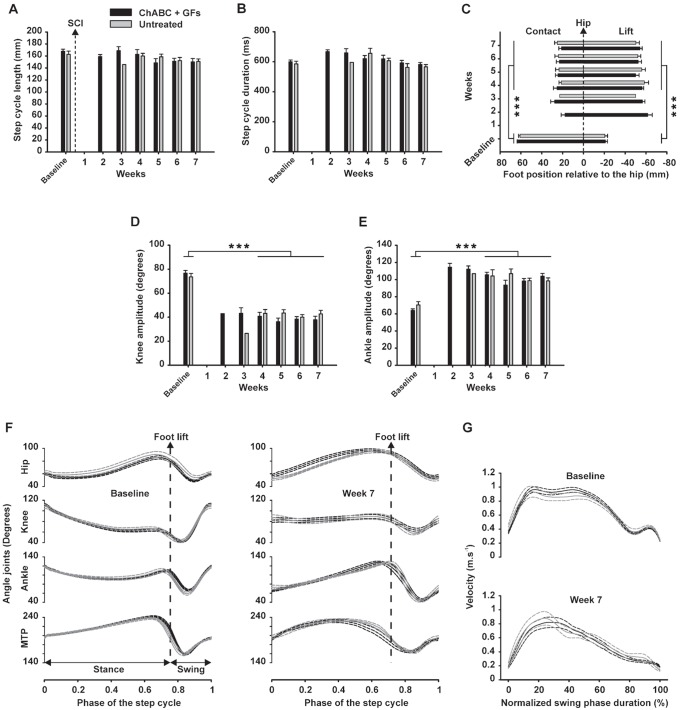
Kinematic analysis of locomotor patterns during the recovery period. Kinematic data were gathered in two groups (i.e. ChABC+GFs and vehicle groups) and averaged before (baseline) and each week for 7 weeks after SCI. Because too few animals were capable to walk on treadmill during the three first weeks after SCI, statistical analysis were performed on baseline and week 4–7 only. (**A**) Mean length of the full step cycle (*i.e.* stance + swing phases) in millimeters is presented. (**B**) Mean duration of the full step cycle in milliseconds are shown. (**C**) Position of the foot contact (*i.e.* left part of the chart) and lift (*i.e.* right part) in millimeters relative to the vertical projection of the great trochanter are depicted (*i.e.* named hip in the chart and represented by the zero value). (**D**) Averaged amplitude of the knee joint in degrees and (**E**) averaged amplitude of ankle joint are presented. (**F**) Averaged angle excursions of the hip, knee, ankle and MTP, before (left panel) and 7 weeks after SCI (right panel) are shown. (**G**) Comparison of the averaged instant foot velocity (*i.e.* full lines) during swing phase before (top panel) and 7 weeks after SCI (bottom panel) for both groups, their respective SEM envelopes (*i.e.* dash line) are given. Symbol *** represent a significance threshold ≤0.001.

In accordance with our previous study on trained and untrained adult rats with a severe thoracic spinal cord clip compression [27], most of injured rats in the present study, regardless of treatment group, spontaneously recovered an impressive capability to generate efficient and coordinated hindlimb locomotor movements given the severity of the lesion. Consequently, the additional treadmill training provided during the 7-week recovery period failed to improve the locomotor parameters measured beyond this considerable spontaneous recovery at least at the end of experiment. In addition, none of the rats in the present study achieved full recovery in lateral balance during treadmill locomotion and only few animals recovered partial weight bearing (based on observations of the experimenters). Consequently, the trunk and/or tail of the rats were manually supported during treadmill training and/or kinematic recording sessions throughout the recovery period. The comparison of groups based on kinematic parameters indicated no significant effect of treadmill training throughout the recovery period. Consequently, to increase the statistical power and focus on the potential effects of ChABC + GFs treatment, the rats were then gathered in two groups: 1) ChABC + GFs treated and 2) vehicle treated animals. Figure 6 illustrates kinematic results of locomotor performance on treadmill from both groups before (*i.e.* baseline) and during the 7 weeks following SCI.

The data showed no significant difference between the two groups in the kinematics of locomotion on treadmill at later time-points. Overall, although weight support and lateral balance remained deficient, when the trunks of the rats were supported they were capable of producing a well-defined and coordinated spontaneous locomotor pattern on the treadmill. In fact, throughout the recovery period, the step cycle length and duration were similar to the baseline (no significant difference; Fig. 6A and B). Interestingly, when the tail and/or trunk support provided by the experimenters was removed, the performance of the rats decreased drastically, they fell on the ground and they were then no longer capable of following the treadmill belt. This observation suggests that after severe compressive SCI, the absence of external postural adjustment, such as during free overground locomotion, could mask the capability of the spinal cord to produce full locomotor pattern.

Although the step cycle length was near-normal at week 7 (Fig. 6A), the parts of the locomotor cycle in front and in back of the vertical projection of the hip was respectively lower (*p*≤0.001; Fig. 6C left part) and higher (*p*≤0.001; Fig. 6C right part). As we reported previously [27], these data demonstrate that the locomotor movements of the hindlimbs shifted backwards. Nevertheless, despite the postural support, the knee joint movement remained affected 7 weeks after SCI (see knee angle excursion in Fig. 6F) and its amplitude was half of the baseline value all along the recovery period (*p*≤0.001; Fig. 6D). To maintain the amplitude of the whole locomotor pattern, the animals compensated by increasing the ankle amplitude in similar proportion than the decrease of the knee (*p*≤0.001; Fig. 6E). The angular excursions of the hindlimbs joints (*i.e.* hip, knee, ankle and MTP) during locomotion were very similar between treated and untreated groups 7 weeks after SCI (Fig. 6F right panel). In addition, though the amplitude of knee and ankle changed (Fig. 6D and E) the global shape of angles joints excursion during locomotion returned close to normal at the end of the post-lesion period (Fig. 6F, right panel compared to the left).

The shape of hindlimb instant velocity during the swing phase was changed 7 weeks after SCI. Even if the acceleration and the peak of velocity in the first 20% of the swing were close to normal (comparison of the first 20% of the normalized swing phase duration between top and bottom panel in Fig. 6G), the injured rats decelerated shortly after (from about 30%; Fig. 6G bottom panel) while normal rats maintained the maximum speed until about 45% of the swing (Fig. 6G top panel). These findings suggest that although the injured rats were capable of reaching the highest velocity of a normal swing phase (*i.e.* about 0.9 m.s^−1^); they lost the ability to maintain this speed. In normal rats, the rebound of velocity in the last 20% of the swing corresponds to the final ankle extension just before the foot landing (Fig. 6G top panel). In injured rats, this rebound is lost 7 weeks after SCI (Fig. 6G bottom panel) due to the incapability of landing the foot far enough in front of the hip vertical projection as shown in the figure 6C.

### Recovery of locomotor coordination

**Figure 7:**
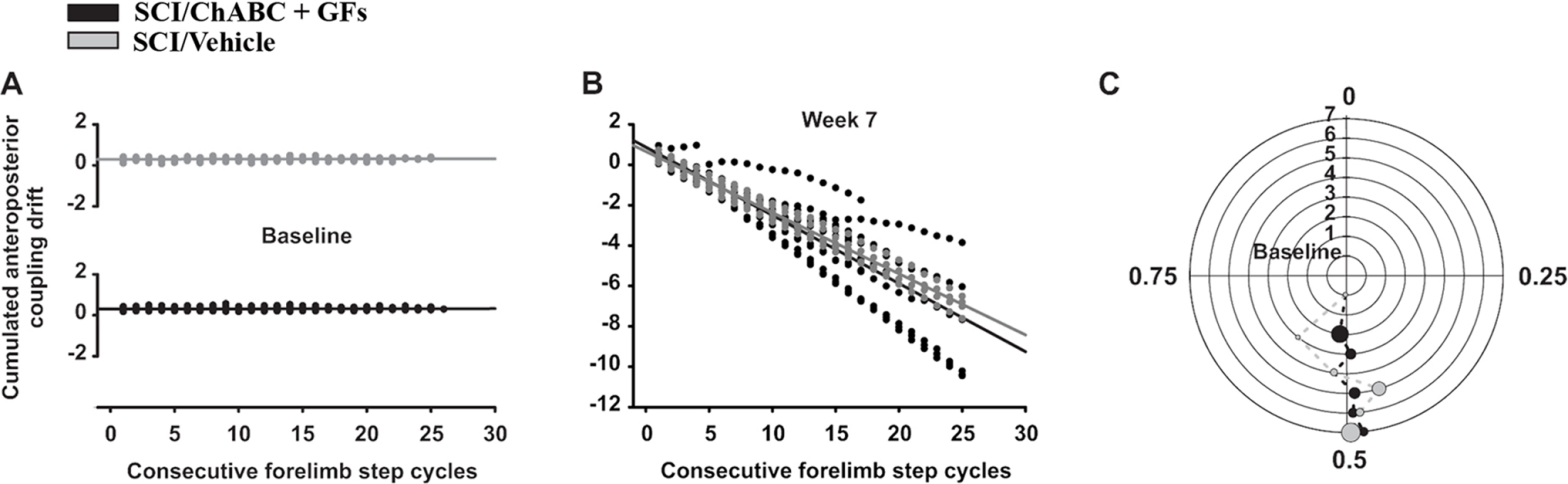
Limb coordination during locomotion. In **A** and **B**, coordination values plotted for each consecutive forelimb step cycle before and 7 weeks after SCI respectively. Usually the coordination measurement is expressed using polar coordinates. To simplify the representation of the coordination drift we converted these data to fit the Cartesian model by subtracting 1 to the polar values when two contacts of the forelimb with the treadmill belt occurred during one hindlimb step cycle (*i.e.* different stepping frequencies). This method renders an account of the intensity of the drift represented by the slope of the consecutive plots. (**C**) Hindlimbs coordination is expressed between 0 and 1 (*i.e.* theta) in a polar plot. The polar axis represents the delays from baseline (*i.e.* the innermost circle) to week 7 (*i.e.* the outermost) post-SCI. Circumference of the circles represent the normalized duration of right step cycle while the dots position on each circle represent the relative time position of the left foot contact averaged by group (see Alluin et al., 2011 for details). In addition, the size of each dot is proportional to the polar dispersion.

SCI rats in both ChABC + GFs and vehicle treated groups recovered the capability of generating locomotor pattern with the hindlimbs; however, they never recovered coupling between anterior and posterior paws while the coordination between both hindlimbs remained similar to the baseline (Figure 7). In normal rats, the frequency of each of the four limbs is the same during locomotion and the anteroposterior coordination value remains very stable around 0.3 all along the treadmill locomotor session (Fig. 7A). After SCI, the coupling between anterior and posterior girdles was lost and unrecovered for all rats all along the post-injury period. This is demonstrated by the progressive drift of the cumulated coordination value during treadmill locomotor sequence at the end of the recovery period (Fig. 7B). In fact, this continuous drift was due to the de-synchronization of the fore- and hindlimbs frequencies after SCI suggesting the loss of neuronal coupling between corresponding CPGs. However, although some fluctuations around the baseline value (*i.e.* 0.5) were present during the post-SCI period, rats have recovered the coordination between both hindlimbs 7 weeks after SCI (Fig. 7C).

### Distribution of walking rats and quality of the locomotor pattern

**Figure 8:**
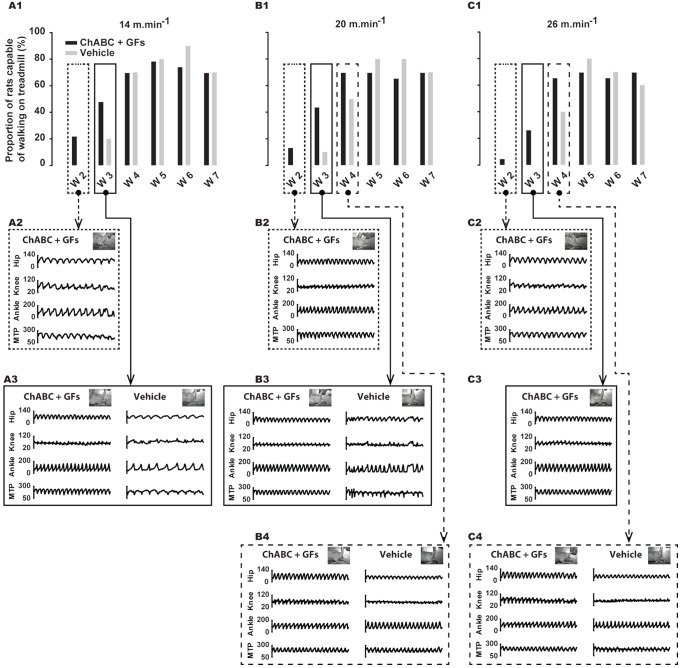
Frequency of rats that have recovered locomotion on treadmill at the three studied velocities and at the different time points throughout the recovery period. (**A1**, **B1** and **C1**), percent of rats capable of walking on treadmill at 14, 20 and 26 m.min^−1^ respectively, before and each week of the recovery period for ChABC+GFs and Vehicle groups. (**A2** and **A3**), raw angular excursions of hip, knee, ankle and MTP joints extracted from representative rats walking at 14 m.min^−1^ at week 2 and 3 respectively. (**B2**, **B3** and **B4**), raw angular excursions of hip, knee, ankle and MTP joints extracted from representative rats walking at 20 m.min^−1^ at week 2, 3 and 4 respectively. (**C2**, **C3** and **C4**), similar to B2, B3 and B4 for 26 m.min^−1^. Data in A1, B1 and C1 are expressed in percentage of the group while the other panels below show angular data expressed in degrees.

Data related to the distribution of rats capable to walk on treadmill at different time points show a trend of an earlier recovery in ChABC + GFs group for the three studied velocities 14, 20 and 26 m.min^−1^ (Fig. 8). Two weeks after SCI, only rats from ChABC + GFs group were capable of walking on the treadmill at all velocities (14 m.min^−1^: 21.73%; 20 m.min^−1^: 13.04%; 26 m.min^−1^: 4.34%; Fig. 8A1, B1 and C1). Three weeks after SCI, the distribution of walking rats was still greater in the treated group at 14 m.min^−1^ (ChABC: 47.82%; Vehicle: 20%: Fig. 8A1) and 20 m.min^−1^ (ChABC: 43.47%; Vehicle: 10%: Fig. 8B1) while only rats from treated group were capable to walk on treadmill at 26 m.min^−1^ (26.08%; Fig. 8C1). Four weeks after SCI the distribution of rats capable of walking on treadmill was similar between groups for the less challenging speed (14 m.min^−1^; Fig. 8A1). However, the proportion of walking rats 4 weeks post-lesion remained greater in ChABC + GFs treated group for the highest speeds, 20 m.min^−1^ (ChABC + GFs: 69.56%; Vehicle: 50%; Fig. 8B1) and 26 m.min^−1^ (ChABC + GFs: 65.21%; Vehicle: 40%; Fig. 8C1). In addition, the comparison of angular excursions of the hip, knee, ankle and MTP from representative rats 3 weeks after the lesion shows clearly that the gait of ChABC + GFs treated rats was more regular and consequently the locomotor patterns was more efficient at 14 and 20 m.min^−1^ (Fig. 8A3 and B3). In addition, at the same period, the ChABC + GFs treated rats still showed a very regular locomotor pattern at the highest and most challenging speed (Fig. 8C3). Four weeks following SCI, although the distribution of walking rats remained in favor of ChABC + GFs treated group at 20 and 26 m.min^−1^ (Fig. 8B1 and C1), the organization of angular patterns became equivalent throughout the groups (Fig. 8B4 and C4).

### Relationship between neuroanatomical parameters and kinematic performance

**Figure 9:**
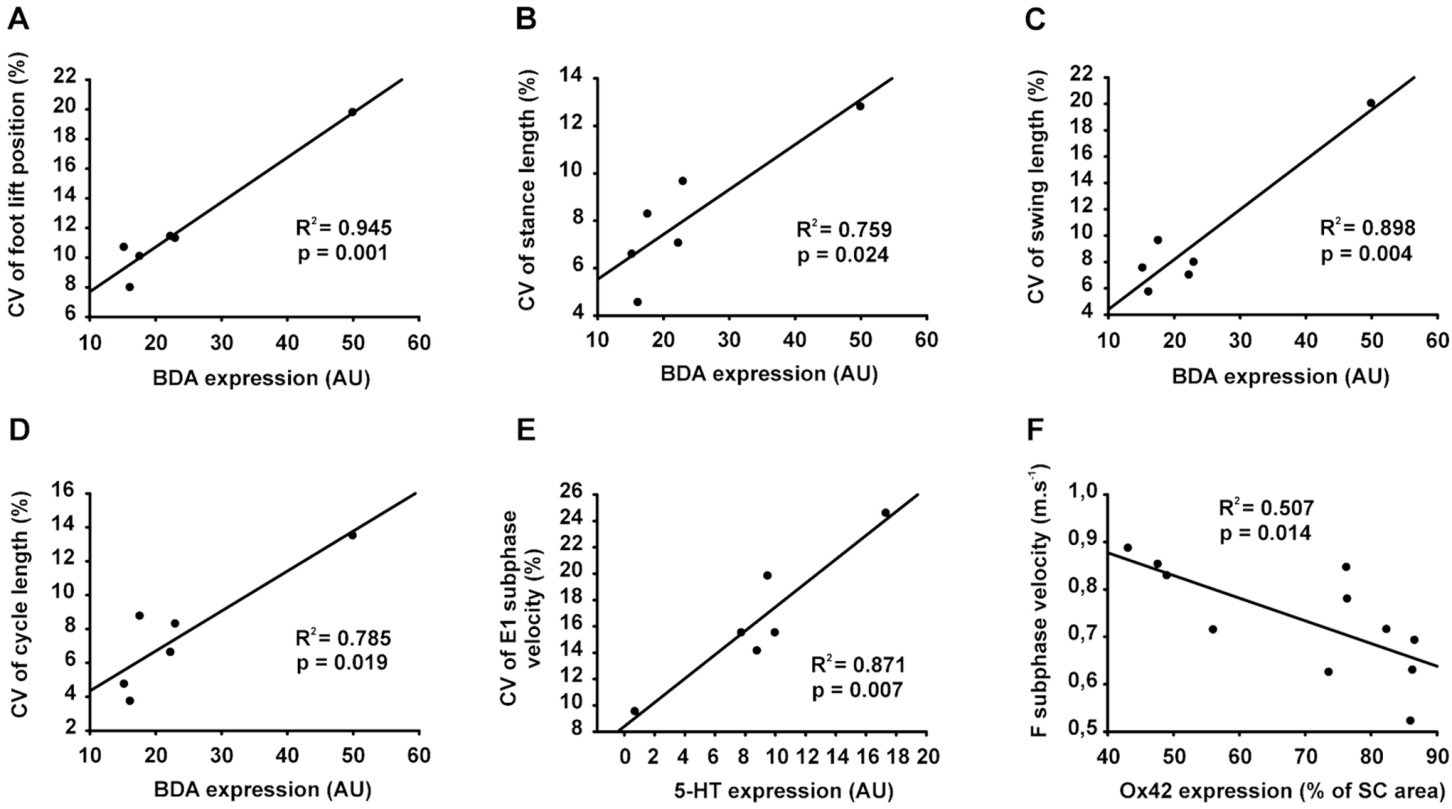
Relationships between behavior and neuroanatomical parameters. Given the absence of significant difference between groups for the behavioral features, available data from all rats were plotted together regardless of the groups. Given that the tissue preparation was different from one labelling protocol to the other, some animals were blindly selected in each group for each different procedure. In addition, among these selected animals not all have recovered the locomotion 7 weeks after SCI (as shown in Fig. 9 A1, B1 and C1). Taken together, this double restriction explains the limited number of plotted data in the present figure. (**A**) Global expression of BDA labeling from the spinal cord section studied was plotted against the coefficient of variation (CV) of the foot position at the onset of the swing phase. (**B**) Graph depicts overall BDA expression against the CV of stance phase length. (**C**) Overall BDA expression against the CV of swing phase length is shown. (**D**) Overall BDA expression against the CV of the step cycle length is depicted. (**E**) Graph shows overall 5-HT expression plotted against the CV of E1 subphase (*i.e.* 2^nd^ part of the swing phase: extension of the hindlimb before foot contact) velocity. (**F**) Overall Ox42 expression is shown against averaged velocity of the F subphase (*i.e.* 1^st^ part of the swing phase: initial flexion of the hindlimb following the foot lift). BDA and 5-HT quantifications are expressed in arbitrary unit (AU), CV is expressed in percentage of the mean and Ox42 labeling is expressed in percentage of the total spinal cord (SC) area. The coefficient of determination (R^2^) and statistical significance (p) are given on each panel.

Since the kinematic data showed no significant effect of treatments on treadmill locomotor performances, the global regression analysis was performed by combining the data from all groups. This approach aimed to highlight the main relationships between behavioural outcomes and various neuroanatomical indices of spinal cord tract integrity at the end of experiment. Figure 9 depicts the significant relationships of kinematic parameters with the overall density of corticospinal tract (CST) fibers (BDA; Fig. 9A–D), serotonergic fibers (5-HT; Fig. 9E) and presence of macrophages/microglia cells (OX42; Fig. 9F) in the injured spinal cord. Our analyses showed that density of the CST fibers in the injured spinal cord was positively correlated with the variability of the foot position at the onset of the swing (*i.e.* foot lift; Fig. 9A). The feet lift occurring at the transition between stance and swing phases, and the length variability of both phases were also positively correlated with the presence of CST fibers (Fig. 9B–C). Consequently, the inconsistent foot lift position could explain the positive relationship between the presence of CST fibers and the variability of the whole step cycle length (Fig. 9D). Similarly, 5-HT expression in the SCI site was positively correlated with the variability of the foot velocity in the last part of the swing phase (*i.e.* hindlimb extension part named E1 subphase; Fig. 9E). Taken together, these results strongly suggest that the change in the gait variability measured over time in the present study represents a plasticity mechanism mediated at least in part by the corticospinal tract whereby there is an attempt to constantly adapt the step cycle compared to a more repetitive robot-like locomotor pattern mainly controlled by spinal circuits. Interestingly, the presence of OX42-positive macrophages/microglia, which is representative of the extent of inflammation, and the foot velocity in the first part of the swing phase (*i.e.* hindlimb flexion part named F subphase) were negatively interrelated at the end of experiment (Fig. 9F). These data suggest that the increase of inflammation in the spinal cord could be related to the decrease in movement velocity especially during swing phase.

## Discussion

In the present study, we investigated the impact of combining daily motor training on treadmill with pharmacological treatment consisting of ChABC as well as growth factors (GFs), containing EGF, bFGF and PDGF-AA, on the structural plasticity of the spinal cord and the recovery of locomotion in rats with clip compression spinal lesion at T7. We report evidence of neuroanatomical spinal plasticity with the combined therapy and modest neurobehavioural effects as exhibited by a trend for an earlier (3–4 weeks after SCI) return of bilateral hindlimb locomotion after SCI in animals treated with ChABC + GFs. However, this combinatorial approach did not induce long-term functionally significant improvement of our specific kinematic parameters at the end of the 7-week recovery period.

### Combinatorial treatment enhances neuroanatomical plasticity in the spinal cord

The use of ChABC and GFs therapy was based on our previous work that showed promising results when combined with neural precursor cell (NPCs) transplantation in promoting neuroplasticity and remyelination in chronic SCI [11]. ChABC and GFs therapy also enhanced endogenous oligodendrocyte replacement and attenuated astrocyte generation and pre-lesional glial scarring and inflammation when applied subacutely following SCI [15]. Our goal in the present study was to determine whether addition of a regular motor training regimen, as a clinically viable therapy, to the ChABC and GFs treatment would synergistically improve the recovery of locomotion after SCI by enhancing activity dependent plasticity in spinal circuitry. On day four after SCI, we delivered ChABC and GFs intrathecally for one week. This delayed administration was specifically chosen for ChABC in SCI since: 1) CSPGs are highly upregulated and easily visualized within the spinal lesion at this time [15], [40], 2) previous studies have shown the beneficial immunomodulatory effects of CSPGs in the acute phase of SCI [41], and 3) delayed therapies would facilitate a clinically relevant therapeutic time window of intervention for SCI. Our findings demonstrate that combining ChABC, GFs and daily exercise synergistically enhanced axonal plasticity in the descending CST and 5-HT spinal cord pathways. Additionally, the pharmacological treatment attenuated the evolution of astrocytic scar formation and chronic inflammation after SCI with a synergistic effect provided by treadmill training. These results are in general agreement with our previous studies in which we combined ChABC, GFs with NPC transplantation [11]. In fact, ChABC administration itself was sufficient to induce remarkable increase in collateral sprouting and/or preservation of the CST in rostral regions to the injury epicenter as well as sprouting of the serotonergic fibers within the lesional and peri-lesional areas [11]. Our neuroanatomical data here are also consistent with previous work by other groups indicating the overall positive impact of inhibiting CSPGs in improving injury microenvironment for repair and regeneration [16]–[18], [22], [42]–[45].

### Absence of synergistic effects of treadmill training with ChABC and GF treatments on measurements of kinematic parameters at the end of the 7-week recovery period

Although the positive effects of ChABC administration on structural plasticity and regeneration in SCI has been established in the literature, its functional impact on specific motor patterns such as locomotion is still debated particularly in compressive/contusive models of SCI. To date, functional benefits of ChABC in SCI has been mainly achieved in partial transection models [12], [13], [16], [18], [19], [22], [44], and when ChABC was administered in more clinically-relevant models of SCI such as contusion and/or compression models as is the case in our study, its effects on functional recovery was minimal [11], [43], [46], [47]. Recently, a study reported the transplantation of genetically modified Schwann cells to secrete endogenously D15A (neurotrophin mimicking the effects of NT-3 and BDNF) and ChABC in the injury site of rats with contusive SCI [21]. In agreement with our present and previous investigations [11], the data of this work showed a strong effect of the modified Schwann cells on the structural plasticity of the spinal cord (axonal regeneration, decrease of CSPGs, increase of myelination and 5-HT positive axons…) but a very moderate effect on the locomotor recovery (+0.5 improvement on the BBB scale compared to control group at the end of the experimental series lasting up to 14 weeks). Functional recovery with ChABC also seems to depend on the severity of SCI lesion. Caggiano and colleagues showed the positive effects of ChABC on hindlimb locomotor recovery only in rats with more severe compressive SCI [48]. Interestingly, in the same study, one experimental group with a moderate injury, comparable to the SCI severity in our present work, received no benefits on the recovery of locomotion from ChABC treatment [48].

Although, we show that rats in all experimental conditions had recovered the capability to generate fast, ample, coordinated and well-defined hindlimb locomotor movements on treadmill at the end of the 7 week recovery period and a trend of earlier return of locomotion in pharmacologically treated rats, our statistical analysis failed to detect any significant effects in treadmill trained animals (with or without pharmacological treatment). These results reinforce the conclusions of previous studies that showed the absence of locomotor improvement in trained rats with incomplete SCI [27]–[29], [49]–[51]. Interestingly, Caudle and colleagues report that when the hindlimbs of rats with mid thoracic contusion are immobilized on adapted wheelchair immediately after SCI, the overground locomotor performance of their hindlimbs decreases dramatically and then increases when the wheelchair is removed suggesting a substantial training effect of spontaneous locomotor movements [52]. Consequently, the absence of treadmill training effect in the present study does not disclaim the beneficial effect of treadmill training after SCI but more likely represents the impact of continuous self-training of untrained SCI rats in their cage as previously suggested [26], [27], [49]. It is plausible that cage exercise in untrained and trained animals enhances their locomotor performance until a ceiling threshold is achieved beyond which any added treadmill training remains ineffective. In this context, it would be interesting in further experiment to challenge our combined strategy by increasing the severity of the spinal compression in order to lower the level of spontaneous recovery and reveal the potential benefits that might be concealed by the intrinsic locomotor capabilities. Further studies are required to elucidate the possibility that our combined therapy could improve functional recovery in a more severe compressive SCI with a lower degree of spontaneous locomotor recovery.

Other variables could also influence the outcomes of our training paradigm such as the duration and frequency of treadmill training and/or the optimal delay between SCI and the start of exercise training. An earlier study in spinal rats showed that the degree of locomotor recovery after SCI is correlated with the number of steps executed during each training session [53]. Therefore, it is plausible that the ten minutes duration of daily training in the present study might be insufficient to induce demonstrable activity-dependent functional improvement in clip compression SCI. Another variable is the impact of therapeutic time-window for initiating rehabilitation after SCI. In fact, studies by Smith and colleagues have shown the deleterious effect of swim training in acute phase of SCI on locomotor performance in rats with moderate contusive thoracic SCI [54]. The authors showed that starting training as early as 3 day after SCI could exert adverse effects on the recovery of locomotion by exacerbating the inflammatory response. Further studies are necessary to evaluate the impact of different variables in rehabilitation paradigms in SCI including time-window, frequency and duration of motor training.

### Potential effects of combined ChABC and growth factors on fore-and hindlimbs coupling

Based on the absence of specific results induced by treadmill training, rats in the present study were consequently gathered into two experimental groups regardless of their training status: ChABC+GFs and vehicle treated groups. This was to focus on the effects of pharmacological treatments while increasing the statistical power of this new approach. Our kinematics analyses of ChABC+GFs and vehicle treated groups showed that injured rats generally regained plantar stepping with occasional weight bearing (not measured) and no evidence of forelimb-hindlimb coordination on treadmill (Fig. 7B). This recovery pattern corresponds to a BBB score of 9 to 10 which represents the baseline of our injury model as described previously [6], [11]. However, although the anteroposterior coupling was globally disrupted in all animals of the present study, a subset of the ChABC+GFs treated group showed evidence of shorts bouts of coordinated frequency between forelimbs and hindlimbs (*i.e.* 3–5 consecutive step cycles) during their locomotor sequences 7 weeks after SCI (see few horizontal alignments of 3–5 consecutive black dots between 0–4, 5–8 and 15–20 forelimb step cycles in Fig. 7B). This transient period of coordination could be due to the perturbation of forelimb or hindlimb movements resulting in temporary forelimb-hindlimb synchronization. However, the fact that it was only evident in the ChABC+GFs group raises the potential benefits of the combined ChABC + GF treatment in establishing functional connections between upper and lower segments of the spinal cord.

It is noteworthy to mention that previous studies showing positive effects of ChABC on axonal regeneration and the potential functional connections used open field behavioural tasks to visually assess locomotion including coordination [11]–[13], [19], [22], [44]. In the present study, we used precise measurement of coordination parameters using kinematics to assess fine locomotor improvements, which are normally unobservable or difficult to assess in open field evaluations. Given this, it is possible that moderate improvements in anteroposterior coupling may have occurred after ChABC treatment that had remained undetected in previous investigations. It is also possible that false positive anteroposterior coupling was observed on a short-term scale. Nonetheless, in the present study, transient occurrences of anteroposterior coupling detected in ChABC+GFs treated group did not reach statistical significance. Future studies involving detailed analysis of interlimb coordination in a greater number of animals are essential to clarify the potential effect of ChABC+GFs treatment on forelimb-hindlimb coupling during locomotion in rat compressive SCI.

### Some evidence of early locomotor recovery induced by the ChABC and GF treatments

Interestingly, our data on the percentage of rats capable of walking on treadmill at different time points after SCI, revealed not only a trend for an earlier recovery of locomotion in ChABC + GFs treated group, but also the improvement of stepping regularity and quality at early stages after SCI as demonstrated by comparison of raw angular excursions from representative rats in both groups in Figure 8. Although these data did not reach statistical significance using our experimental design, the persistent trends at several consecutive time points and velocities suggest a possible effect of combined pharmacological treatment on the spinal cellular mechanisms early after the lesion. Immediately after SCI, influx of the inflammatory cells as well as the spinal shock occurs that targets the motoneurons below the lesion resulting in immediate loss of hindlimb movements and reflexes. This initial spinal shock is then followed by a gradual recovery of reflexes and, depending of the severity of lesion, regain of some motor functions. In the present study, we show a significant decrease in the presence of OX42 (CD11b) expressing macrophages/microglia in ChABC+GFs treated rats compared to vehicle counterparts (Fig. 3) suggesting the immunomodulatory effects of ChABC+GFs treatment on the injury induced-inflammatory response. Decrease in the inflammatory response in ChABC+GFs treated group may suggest a reduced period of spinal shock in these animals that could result in an earlier return of locomotor function observed in some of these animals. Here also, further investigations are needed to delineate the modulatory roles of these treatments on the recruitment of inflammatory cells as well as the impact of neuroinflammation inhibition on the time course of locomotor recovery after SCI.

### Greater gait variability after SCI as a sign of greater supraspinal influence?

The correlation between the quantitative histological assessments and variability in the kinematic readouts is of interest. Such variability could be interpreted to mean that the step cycles are simply variable in nature or that the neuroplasticity induced by our treatment is aberrant and disrupting but it could also mean that the variability in the kinematic parameters reflect greater adaptability of these readouts. For instance, in a robotic locomotor performance in which the variability is dictated by the immediate environment only, such an automatic rhythmic behavior could be considered at the end of a spectrum where locomotion is principally defined by the operation of a spinal circuitry. However, it is considered that after partial SCI there are descending fibers re-innervating the spinal cord in an attempt to impose correcting inputs to the spinal cord. This might lead to a seemingly disorganized pattern but a pattern towards optimizing the motor performance despite the spinal lesion. We presume that the greater variability of the locomotor pattern may represent an attempt at a much more long-term functional recovery of the spinal cord. For instance, we can surmise that some degree of incoordination between the fore- and hindlimbs, as observed in the present study, might require adaptive corrections leading to a more apparent variability in the hindlimb step cycles. Our previous work in cats involved in a dual spinal lesion paradigm indicates that kinematic parameters of complete spinal cats are less variable than of hemisected cats suggesting that remnant descending pathways play a role in attempting to regulate the step cycle [55]. Similarly, in the present rat experiments, it could be postulated that as more supraspinal inputs reach the spinal cord through plastic mechanisms, the more variable step cycles may be represent as an attempt to continuously adapt the step cycles to the supraspinal inputs.

On the other hand, the recovery of hindlimb locomotion after partial spinal lesions depends also on intrinsic changes within the spinal cord itself. Indeed, several experiments in cats have shown that, after a thoracic hemisection, the spinal cord below the spinal lesion is durably changed since, after a further complete spinal section, the spinal cord can immediately express hindlimb locomotion whereas it usually takes 2–3 weeks of training to achieve such level of performance [56]–[59]. That such changes do occur in the spinal cord itself has consequences on the overall locomotor behavior of the animal since remnant inputs will encounter a more excitable spinal cord that has been changed by the previous lesion. In the present study, the large spinal lesion produced by the compression also leaves a great deal of autonomy to the spinal cord below the crush. It is therefore likely that such intrinsic spinal mechanisms also take place and those changes in intrinsic circuitry and neuroanatomical plasticity occurring below the lesion are part of the mechanisms leading to a seemingly faster optimal recovery of the hindlimb locomotor behavior [27].

### Limitations and stringency of our behavioral assessment

Here, we assessed locomotor parameters on a treadmill but not as such changes in posture, in reflex transmission or in autonomic functions and it could be that some of the neuroanatomical changes seen reflect modifications of these parameters. Furthermore, changes in neuroanatomy and function may not be necessarily synergistic as seen here and in previous studies in which combined treatment of anti-Nogo-A antibody and training showed different beneficial effects but not synergistic [60]. Moreover, all parameters of improvement at a given time point may not reflect all the underlying processes that might be controlled by intrinsic neural mechanisms as well as environmental mechanisms such as provided by locomotor training. Thus, locomotor recovery after SCI can be defined from several points of views and with different degree of precision. Precise kinematic analyses such as employed here may be a double-edge sword. They provide an objective measurement of some specific parameters (step length for instance) but may fail to provide a complete global depiction of locomotion since not all kinematic parameters can be considered all the time at various speeds and epochs after SCI. Furthermore, the continuous scrolling of the belt during treadmill locomotion may provide afferent feedbacks from the hindlimbs, stimulating the locomotor system to generate locomotor movements and probably change the balance between spinal and supraspinal contributions to the control of locomotion decreasing the role of the supraspinal control while increasing that of the spinal circuitry.

## Conclusions

Our findings demonstrate the beneficial effects of combined ChABC, growth factors and locomotor training on enhancing structural plasticity of the injured cord and despite an impressive spontaneous locomotor recovery observed in our model; we report modest neurobehavioral improvement in treated animals. However, the lack of significant kinematic evidence of sustained functional improvement beyond the spontaneous recovery, at least using our stringent video-based field-by-field analysis, suggests that additional approaches such as cell therapies and/or more appropriate locomotor training may be needed to optimize this therapeutic strategy.

## References

[1] Sekhon LH, Fehlings MG (2001) Epidemiology, demographics, and pathophysiology of acute spinal cord injury. Spine 26: S2–12. 11805601 10.1097/00007632-200112151-00002

[2] Tator CH (1998) Biology of Neurological Recovery and Functional Restoration after Spinal Cord Injury. Neurosurgery 42: 696–708. 9574633 10.1097/00006123-199804000-00007

[3] van Hedel HJ, Dietz V (2010) Rehabilitation of locomotion after spinal cord injury. Restor Neurol Neurosci 28: 123–134. 20086289 10.3233/RNN-2010-0508

[4] Wilson JR, Grossman RG, Frankowski RF, Kiss A, Davis AM, et al (2012) A clinical prediction model for long-term functional outcome after traumatic spinal cord injury based on acute clinical and imaging factors. J Neurotrauma 29: 2263–2271. 22709268 10.1089/neu.2012.2417 PMC3430477

[5] Fawcett JW, Curt A, Steeves JD, Coleman WP, Tuszynski MH, et al (2007) Guidelines for the conduct of clinical trials for spinal cord injury as developed by the ICCP panel: spontaneous recovery after spinal cord injury and statistical power needed for therapeutic clinical trials. Spinal Cord 45: 190–205. 17179973 10.1038/sj.sc.3102007

[6] Karimi-Abdolrezaee S, Eftekharpour E, Wang J, Morshead C, Fehlings M (2006) Delayed Transplantation of Adult Neural Stem Cells Promotes Remyelination and functional recovery after Spinal Cord Injury. The Journal of Neuroscience, March 29 26(13): 3377–3389. 10.1523/JNEUROSCI.4184-05.2006 PMC6673854 16571744

[7] Fouad K, Dietz V, Schwab ME (2001) Improving axonal growth and functional recovery after experimental spinal cord injury by neutralizing myelin associated inhibitors. Brain Res Brain Res Rev 36: 204–212. 11690617 10.1016/s0165-0173(01)00096-0

[8] Keirstead HS, Nistor G, Bernal G, Totoiu M, Cloutier F, et al (2005) Human embryonic stem cell-derived oligodendrocyte progenitor cell transplants remyelinate and restore locomotion after spinal cord injury. J Neurosci 25: 4694–4705. 15888645 10.1523/JNEUROSCI.0311-05.2005 PMC6724772

[9] Lu P, Wang Y, Graham L, McHale K, Gao M, et al (2012) Long-distance growth and connectivity of neural stem cells after severe spinal cord injury. Cell 150: 1264–1273. 22980985 10.1016/j.cell.2012.08.020 PMC3445432

[10] Lu P, Blesch A, Graham L, Wang Y, Samara R, et al (2012) Motor axonal regeneration after partial and complete spinal cord transection. J Neurosci 32: 8208–8218. 22699902 10.1523/JNEUROSCI.0308-12.2012 PMC3407545

[11] Karimi-Abdolrezaee S, Eftekharpour E, Wang J, Schut D, Fehlings MG (2010) Synergistic effects of transplanted adult neural stem/progenitor cells, chondroitinase, and growth factors promote functional repair and plasticity of the chronically injured spinal cord. J Neurosci 30: 1657–1676. 20130176 10.1523/JNEUROSCI.3111-09.2010 PMC6634011

[12] Bradbury EJ, Moon LD, Popat RJ, King VR, Bennett GS, et al (2002) Chondroitinase ABC promotes functional recovery after spinal cord injury. Nature 416: 636–640. 11948352 10.1038/416636a

[13] Garcia-Alias G, Petrosyan HA, Schnell L, Horner PJ, Bowers WJ, et al (2011) Chondroitinase ABC combined with neurotrophin NT-3 secretion and NR2D expression promotes axonal plasticity and functional recovery in rats with lateral hemisection of the spinal cord. J Neurosci 31: 17788–17799. 22159095 10.1523/JNEUROSCI.4308-11.2011 PMC3758578

[14] Fouad K, Schnell L, Bunge MB, Schwab ME, Liebscher T, et al (2005) Combining Schwann cell bridges and olfactory-ensheathing glia grafts with chondroitinase promotes locomotor recovery after complete transection of the spinal cord. J Neurosci 25: 1169–1178. 15689553 10.1523/JNEUROSCI.3562-04.2005 PMC6725952

[15] Karimi-Abdolrezaee S, Schut D, Wang J, Fehlings MG (2012) Chondroitinase and growth factors enhance activation and oligodendrocyte differentiation of endogenous neural precursor cells after spinal cord injury. PLoS One 7: e37589. 22629425 10.1371/journal.pone.0037589 PMC3358255

[16] Wang D, Ichiyama RM, Zhao R, Andrews MR, Fawcett JW (2011) Chondroitinase combined with rehabilitation promotes recovery of forelimb function in rats with chronic spinal cord injury. J Neurosci 31: 9332–9344. 21697383 10.1523/JNEUROSCI.0983-11.2011 PMC6623473

[17] Massey JM, Hubscher CH, Wagoner MR, Decker JA, Amps J, et al (2006) Chondroitinase ABC digestion of the perineuronal net promotes functional collateral sprouting in the cuneate nucleus after cervical spinal cord injury. J Neurosci 26: 4406–4414. 16624960 10.1523/JNEUROSCI.5467-05.2006 PMC6673998

[18] Tom VJ, Kadakia R, Santi L, Houle JD (2009) Administration of chondroitinase ABC rostral or caudal to a spinal cord injury site promotes anatomical but not functional plasticity. J Neurotrauma 26: 2323–2333. 19659409 10.1089/neu.2009.1047 PMC2824222

[19] Lee H, McKeon RJ, Bellamkonda RV (2010) Sustained delivery of thermostabilized chABC enhances axonal sprouting and functional recovery after spinal cord injury. Proc Natl Acad Sci U S A 107: 3340–3345. 19884507 10.1073/pnas.0905437106 PMC2840440

[20] Garcia-Alias G, Barkhuysen S, Buckle M, Fawcett JW (2009) Chondroitinase ABC treatment opens a window of opportunity for task-specific rehabilitation. Nat Neurosci 12: 1145–1151. 19668200 10.1038/nn.2377

[21] Kanno H, Pressman Y, Moody A, Berg R, Muir EM, et al (2014) Combination of engineered Schwann cell grafts to secrete neurotrophin and chondroitinase promotes axonal regeneration and locomotion after spinal cord injury. J Neurosci 34: 1838–1855. 24478364 10.1523/JNEUROSCI.2661-13.2014 PMC3905147

[22] Alilain WJ, Horn KP, Hu H, Dick TE, Silver J (2011) Functional regeneration of respiratory pathways after spinal cord injury. Nature 475: 196–200. 21753849 10.1038/nature10199 PMC3163458

[23] Carter LM, McMahon SB, Bradbury EJ (2011) Delayed treatment with chondroitinase ABC reverses chronic atrophy of rubrospinal neurons following spinal cord injury. Exp Neurol 228: 149–156. 21215745 10.1016/j.expneurol.2010.12.023

[24] Barritt AW, Davies M, Marchand F, Hartley R, Grist J, et al (2006) Chondroitinase ABC promotes sprouting of intact and injured spinal systems after spinal cord injury. J Neurosci 26: 10856–10867. 17050723 10.1523/JNEUROSCI.2980-06.2006 PMC3339436

[25] Bukhari N, Torres L, Robinson JK, Tsirka SE (2011) Axonal regrowth after spinal cord injury via chondroitinase and the tissue plasminogen activator (tPA)/plasmin system. J Neurosci 31: 14931–14943. 22016526 10.1523/JNEUROSCI.3339-11.2011 PMC3206287

[26] Jakeman LB, Hoschouer EL, Basso DM (2011) Injured mice at the gym: review, results and considerations for combining chondroitinase and locomotor exercise to enhance recovery after spinal cord injury. Brain Res Bull 84: 317–326. 20558254 10.1016/j.brainresbull.2010.06.002 PMC3030989

[27] Alluin O, Karimi-Abdolrezaee S, Delivet-Mongrain H, Leblond H, Fehlings MG, et al (2011) Kinematic study of locomotor recovery after spinal cord clip compression injury in rats. J Neurotrauma 28: 1963–1981. 21770755 10.1089/neu.2011.1840

[28] Fouad K, Metz GA, Merkler D, Dietz V, Schwab ME (2000) Treadmill training in incomplete spinal cord injured rats. Behav Brain Res 115: 107–113. 10996413 10.1016/s0166-4328(00)00244-8

[29] Hutchinson KJ, Gomez-Pinilla F, Crowe MJ, Ying Z, Basso DM (2004) Three exercise paradigms differentially improve sensory recovery after spinal cord contusion in rats. Brain 127: 1403–1414. 15069022 10.1093/brain/awh160

[30] Gauthier MK, Kosciuczyk K, Tapley L, Karimi-Abdolrezaee S (2013) Dysregulation of the neuregulin-1-ErbB network modulates endogenous oligodendrocyte differentiation and preservation after spinal cord injury. Eur J Neurosci. 10.1111/ejn.12268 23758598

[31] Karimi-Abdolrezaee S, Eftekharpour E, Fehlings MG (2004) Temporal and spatial patterns of Kv1.1 and Kv1.2 protein and gene expression in spinal cord white matter after acute and chronic spinal cord injury in rats: implications for axonal pathophysiology after neurotrauma. Eur J Neurosci 19: 577–589. 14984408 10.1111/j.0953-816x.2004.03164.x

[32] Fehlings MG, Tator CH (1995) The relationships among the severity of spinal cord injury, residual neurological function, axon counts, and counts of retrogradely labeled neurons after experimental spinal cord injury. Exp Neurol 132: 220–228. 7789460 10.1016/0014-4886(95)90027-6

[33] Eftekharpour E, Karimi-Abdolrezaee S, Wang J, Morshead C, Fehlings M (2007) Myelination of Congenitally Dysmyelinated Spinal Cord Axons by Adult Neural Precursor Cells Results in Formation of Nodes of Ranvier and Improved Axonal Conduction. The Journal of Neuroscience 27: 3416–3428. 17392458 10.1523/JNEUROSCI.0273-07.2007 PMC6672112

[34] Karimi-Abdolrezaee S, Schreyer DJ (2002) Retrograde repression of growth-associated protein-43 mRNA expression in rat cortical neurons. J Neurosci 22: 1816–1822. 11880510 10.1523/JNEUROSCI.22-05-01816.2002 PMC6758898

[35] Seif GI, Nomura H, Tator CH (2007) Retrograde axonal degeneration “dieback” in the corticospinal tract after transection injury of the rat spinal cord: a confocal microscopy study. J Neurotrauma 24: 1513–1528. 17892412 10.1089/neu.2007.0323

[36] Ciranna L (2006) Serotonin as a Modulator of Glutamate- and GABA-Mediated Neurotransmission: Implications in Physiological Functions and in Pathology. Curr Neuropharmacol 4: 101–114. 18615128 10.2174/157015906776359540 PMC2430669

[37] Jordan LM, Liu J, Hedlund PB, Akay T, Pearson KG (2008) Descending command systems for the initiation of locomotion in mammals. Brain Res Rev 57: 183–191. 17928060 10.1016/j.brainresrev.2007.07.019

[38] Schmidt BJ, Jordan LM (2000) The role of serotonin in reflex modulation and locomotor rhythm production in the mammalian spinal cord. Brain Res Bull 53: 689–710. 11165804 10.1016/s0361-9230(00)00402-0

[39] Husch A, Van Patten GN, Hong DN, Scaperotti MM, Cramer N, et al (2012) Spinal cord injury induces serotonin supersensitivity without increasing intrinsic excitability of mouse V2a interneurons. J Neurosci 32: 13145–13154. 22993431 10.1523/JNEUROSCI.2995-12.2012 PMC3506248

[40] Gris P, Tighe A, Levin D, Sharma R, Brown A (2007) Transcriptional regulation of scar gene expression in primary astrocytes. Glia 55: 1145–1155. 17597120 10.1002/glia.20537

[41] Rolls A, Shechter R, London A, Segev Y, Jacob-Hirsch J, et al (2008) Two faces of chondroitin sulfate proteoglycan in spinal cord repair: a role in microglia/macrophage activation. PLoS Med 5: e171. 18715114 10.1371/journal.pmed.0050171 PMC2517615

[42] Busch SA, Horn KP, Silver DJ, Silver J (2009) Overcoming macrophage-mediated axonal dieback following CNS injury. J Neurosci 29: 9967–9976. 19675231 10.1523/JNEUROSCI.1151-09.2009 PMC2771342

[43] Novotna I, Slovinska L, Vanicky I, Cizek M, Radonak J, et al (2011) IT delivery of ChABC modulates NG2 and promotes GAP-43 axonal regrowth after spinal cord injury. Cell Mol Neurobiol 31: 1129–1139. 21630006 10.1007/s10571-011-9714-1 PMC11498576

[44] Starkey ML, Bartus K, Barritt AW, Bradbury EJ (2012) Chondroitinase ABC promotes compensatory sprouting of the intact corticospinal tract and recovery of forelimb function following unilateral pyramidotomy in adult mice. Eur J Neurosci 36: 3665–3678. 23061434 10.1111/ejn.12017 PMC4851235

[45] Fouad K, Pearse DD, Tetzlaff W, Vavrek R (2009) Transplantation and repair: combined cell implantation and chondroitinase delivery prevents deterioration of bladder function in rats with complete spinal cord injury. Spinal Cord 47: 727–732. 19255587 10.1038/sc.2009.10

[46] Mountney A, Zahner MR, Sturgill ER, Riley CJ, Aston JW, et al (2013) Sialidase, chondroitinase ABC, and combination therapy after spinal cord contusion injury. J Neurotrauma 30: 181–190. 22934782 10.1089/neu.2012.2353 PMC3565552

[47] Yang YG, Jiang DM, Quan ZX, Ou YS (2009) Insulin with chondroitinase ABC treats the rat model of acute spinal cord injury. J Int Med Res 37: 1097–1107. 19761692 10.1177/147323000903700414

[48] Caggiano AO, Zimber MP, Ganguly A, Blight AR, Gruskin EA (2005) Chondroitinase ABCI improves locomotion and bladder function following contusion injury of the rat spinal cord. J Neurotrauma 22: 226–239. 15716629 10.1089/neu.2005.22.226

[49] Heng C, de Leon RD (2009) Treadmill training enhances the recovery of normal stepping patterns in spinal cord contused rats. Exp Neurol 216: 139–147. 19111541 10.1016/j.expneurol.2008.11.023 PMC3297429

[50] Ichiyama R, Potuzak M, Balak M, Kalderon N, Edgerton VR (2009) Enhanced motor function by training in spinal cord contused rats following radiation therapy. PLoS One 4: e6862. 19718437 10.1371/journal.pone.0006862 PMC2729923

[51] Kuerzi J, Brown EH, Shum-Siu A, Siu A, Burke D, et al (2010) Task-specificity vs. ceiling effect: step-training in shallow water after spinal cord injury. Exp Neurol 224: 178–187. 20302862 10.1016/j.expneurol.2010.03.008 PMC2885471

[52] Caudle KL, Brown EH, Shum-Siu A, Burke DA, Magnuson TS, et al (2011) Hindlimb immobilization in a wheelchair alters functional recovery following contusive spinal cord injury in the adult rat. Neurorehabil Neural Repair 25: 729–739. 21697451 10.1177/1545968311407519 PMC4419333

[53] Cha J, Heng C, Reinkensmeyer DJ, Roy RR, Edgerton VR, et al (2007) Locomotor ability in spinal rats is dependent on the amount of activity imposed on the hindlimbs during treadmill training. J Neurotrauma 24: 1000–1012. 17600516 10.1089/neu.2006.0233

[54] Smith RR, Brown EH, Shum-Siu A, Whelan A, Burke DA, et al (2009) Swim training initiated acutely after spinal cord injury is ineffective and induces extravasation in and around the epicenter. J Neurotrauma 26: 1017–1027. 19331515 10.1089/neu.2008.0829 PMC2848951

[55] Barriere G, Frigon A, Leblond H, Provencher J, Rossignol S (2010) Dual spinal lesion paradigm in the cat: evolution of the kinematic locomotor pattern. J Neurophysiol 104: 1119–1133. 20573971 10.1152/jn.00255.2010

[56] Barriere G, Leblond H, Provencher J, Rossignol S (2008) Prominent role of the spinal central pattern generator in the recovery of locomotion after partial spinal cord injuries. J Neurosci 28: 3976–3987. 18400897 10.1523/JNEUROSCI.5692-07.2008 PMC6670475

[57] Martinez M, Delivet-Mongrain H, Leblond H, Rossignol S (2012) Effect of locomotor training in completely spinalized cats previously submitted to a spinal hemisection. J Neurosci 32: 10961–10970. 22875930 10.1523/JNEUROSCI.1578-12.2012 PMC6621008

[58] Martinez M, Delivet-Mongrain H, Rossignol S (2013) Treadmill training promotes spinal changes leading to locomotor recovery after partial spinal cord injury in cats. J Neurophysiol 109: 2909–2922. 23554433 10.1152/jn.01044.2012

[59] Rossignol SS, B.J.; Jordan,L.M (2014) Spinal plasticity underlying the recovery of locomotion after injury. In: Selzer MEC, S.; Cohen,L.G.; Kwakkel,G.; Miller,R.H., editor. Textbook of Neural Repair and Rehabilitation. Cambridge: Cambridge University Press.

[60] Maier IC, Ichiyama RM, Courtine G, Schnell L, Lavrov I, et al (2009) Differential effects of anti-Nogo-A antibody treatment and treadmill training in rats with incomplete spinal cord injury. Brain 132: 1426–1440. 19372269 10.1093/brain/awp085

